# Deep immune cell profiling in blood and bone marrow of early stage monoclonal gammopathy: an iStopMM and ECRIN-M3 collaborative study

**DOI:** 10.1038/s41408-025-01255-3

**Published:** 2025-03-27

**Authors:** Oihane Pérez-Escurza, Juan Flores-Montero, Jón Þórir Óskarsson, Luzalba Sanoja-Flores, Julio Pozo, Quentin Lécrevisse, Silvia Martín, Elín Ruth Reed, Guðlaug Katrín Hákonardóttir, Stephen Harding, Sigrún Þorsteinsdóttir, Sæmundur Rögnvaldsson, Thorvardur Jon Love, Brian Durie, Sigurdur Yngvi Kristinsson, Alberto Orfao, on behalf of the iStopMM and ECRIN-M3 consortia, Guðlaug Katrín Hákonardóttir, Sigrún Þorsteinsdóttir, Sæmundur Rögnvaldsson, Thorvardur Jon Love, Sigurdur Yngvi Kristinsson

**Affiliations:** 1 Translational and Clinical Research Program, Cancer Research Center (IBMCC, CSIC—University of Salamanca); Cytometry Service, NUCLEUS; Department of Medicine, University of Salamanca (Universidad de Salamanca), Salamanca, Spain; 2 Institute of Biomedical Research of Salamanca (IBSAL), Salamanca, Spain; 3 Department of Medicine, University of Salamanca (Universidad de Salamanca), Salamanca, Spain; 4 Biomedical Research Networking Centre Consortium of Oncology—CIBERONC (CB16/12/00400)–, Instituto de Salud Carlos III, Madrid, Spain; 5 Department of Hematology, University Hospital of Salamanca, Salamanca, Spain; 6 Faculty of Medicine, University of Iceland, Reykjavík, Iceland; 7 Institute of Biomedicine of Seville, Department of Hematology, University Hospital Virgen del Rocío of the Consejo Superior de Investigaciones Científicas (CSIC), University of Seville, Seville, Spain; 8 The Binding Site Group, Birmingham, UK; 9 Department of Hematology, Rigshospitalet, Copenhagen, Denmark; 10 Department of Science, Landspitali University Hospital, Reykjavík, Iceland; 11 Department of Hematology and Oncology, Cedars-Sinai Medical Center, Los Angeles, CA USA; 12 Department of Hematology, Landspitali University Hospital, Reykjavík, Iceland

**Keywords:** Translational research, Immunoproliferative disorders, Immunosurveillance, Immunoproliferative disorders, Adaptive immunity

Dear Editor,

Increasing evidences support a key role of the immune system in the development and progression of monoclonal gammopathies (MG), i.e., from premalignant stages—e.g., MG of undetermined significance (MGUS), smoldering multiple myeloma (SMM) and smoldering Waldenström’s macroglobulinemia (SWM)—to overt malignant diseases—e.g., multiple myeloma (MM) and Waldenström’s macroglobulinemia (WM)–[1–3]. In this regard, an altered crosstalk in the bone marrow (BM) between neoplastic cells—clonal plasma cells (cPC) and clonal B-cells (cB-cells)—and the surrounding immune cell populations may lead to an impaired response that favors immunoescape, clonal growth and disease progression [1–3]. However, the specific mechanisms involved in loss of immunosurveillance, together with those immune alterations that emerge at the earliest stages of MGUS, remain largely unknown. Here, we used next-generation flow cytometry (Table S1) to we investigate the distribution of a broad variety of immune cell populations (*n* = 360) in paired blood and BM samples of 75 (otherwise) healthy donors (HD) from the general population who screened positive for MGUS—*n* = 55 (18 IgM-MGUS; 37 non-IgM-MGUS)–, SMM (*n* = 12) and SWM (*n* = 8), within the iStopMM program [4] (Data S1).

Except for increased eosinophil (*p* = 0.01) and decreased dendritic cells (DC) numbers (*p* = 0.02), MGUS cases showed normal blood counts of all other major myeloid cell populations. In parallel, a decreased neutrophil production was found in BM of MGUS (*p* < 0.001), SMM (*p* < 0.001) and SWM (*p* = 0.009), which only translated into abnormally low blood counts at the more advanced disease stages—i.e., SMM (*p* = 0.003)–, in line with previous observations in MM [5]. Unlike neutrophils, an increased percentage of total BM monocytes was observed in MGUS (*p* = 0.02) and SMM (*p* = 0.006) [6], associated with normal total monocyte counts in blood, but decreased levels of non-classical—i.e., CD36^−^ and Slan^+^ in MGUS (*p* ≤ 0.003); CD36^+^ and Slan^-^ in MGUS (*p* ≤ 0.004) and SMM (*p* ≤ 0.008)—monocytes (*p* < 0.001) in MGUS, which suggests a preserved monocyte production in BM associated with a potentially increased migration of blood monocytes to tissues, preferentially affecting the more mature (i.e., non-classical) monocyte compartment [6]. Conversely, SWM patients displayed significantly increased monocyte counts in blood at the expense of the major subsets of classical—CD62L^−^, CD62L^+^ and FcεRI^-^ (*p* ≤ 0.005)—monocytes (*p* ≤ 0.001). In parallel, decreased numbers of blood circulating Axl^+^ DC, associated with lower blood counts of both CD1c^+^ myeloid (m)DC and CD141^+^ mDC, only CD141^+^ mDC, or just plasmacytoid (p)DC, were found in MGUS (*p* ≤ 0.03), SMM (*p* ≤ 0.04) and SWM (*p* ≤ 0.03) subjects, respectively, supporting an increased migration to the tumor sites of Axl^+^ DC [2, 3, 7], together with different subsets of mDC and pDC in MGUS, SMM and SWM, respectively. This might (locally) promote different immune cell microenvironments [2, 8] and (potentially also) unique downstream T-cell and adaptive immune response profiles in MGUS, SMM and SWM, modulated in SWM by locally increased monocytic-myeloid-derived suppressor cells (M-MDSC) (*p* = 0.02) (Fig. 1 and S1; Tables S2-5).

**Fig. 1:**
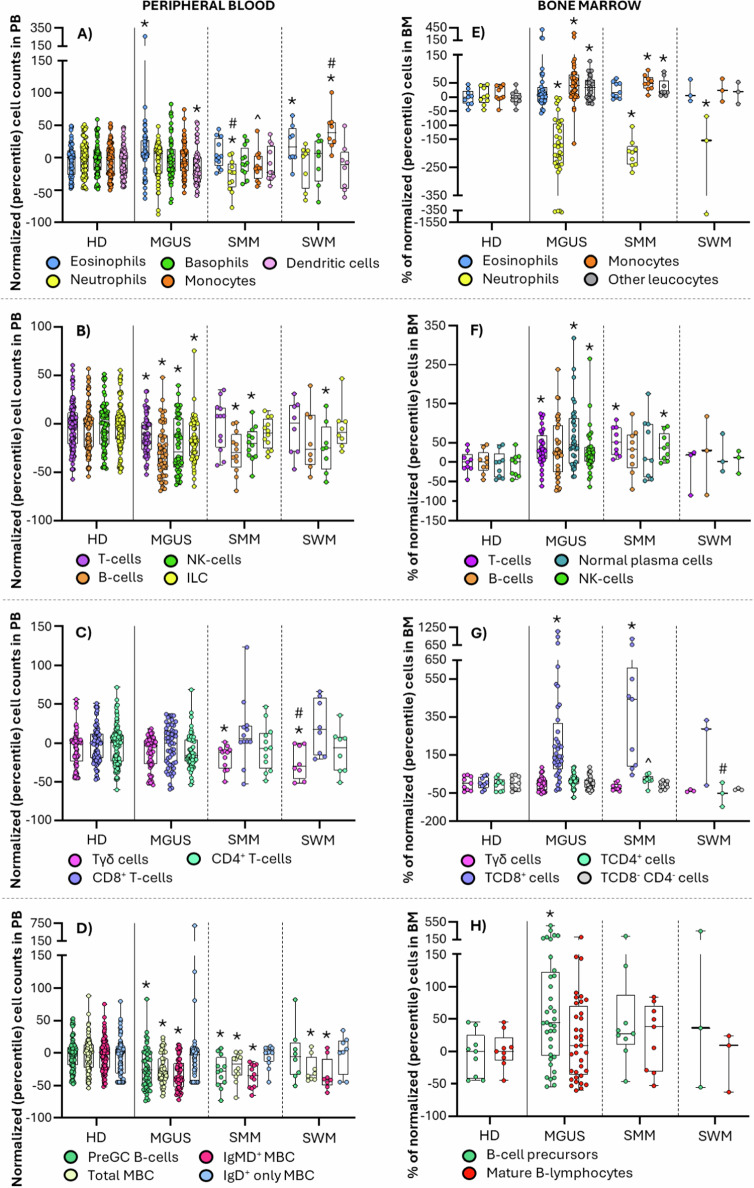
Distribution of the main immune cell populations in blood and BM of monoclonal gammopathy of undetermined significance (MGUS) (*n* = 55), smoldering multiple myeloma (SMM) (*n* = 12) and smoldering Waldenström’s macroglobulinemia (SWM) (*n* = 8) patients compared to healthy donors (HD) (*n* = 118) and normal BM (*n* = 57). In (**A**–**D**), the distribution in peripheral blood of total eosinophils, neutrophils, basophils, monocytes and dendritic cells (**A**), total T-cells, B-cells, NK-cells and ILC (**B**), Tγδ^+^, TCD8^+^ and TCD4^+^ cells (**C**), and pre-germinal center B-cells and IgMD^+^ and IgD^+^ memory B-cells (MBC) (**D**) is shown, while in panels E-H the percentage of bone marrow total eosinophils, neutrophils, monocytes and nucleated red cells (**E**), total T-cells, B-cells, normal plasma cells and NK-cells (F), Tγδ^+^, TCD8^+^, TCD4^+^ and TCD8^-^ CD4^-^ cells (**G**), and pre-germinal center B-cells and mature B-lymphocytes (**H**) is displayed. Box plots extend from the 25th to the 75th percentile values, while the horizontal lines indicate median values and both the minimum and maximum values (whiskers) of cell counts in blood and percentage numbers in bone marrow normalized for each individual case (inner circles) by the median percentile values of aged-matched HD. HD, healthy donor; ILC, innate lymphoid cells; MBC, memory B-cells; MGUS, monoclonal gammopathy of undetermined significance; preGC, pre-germinal center; SMM, smoldering multiple myeloma; SWM, smoldering Waldenström’s macroglobulinemia. *p < 0.05 vs HD; #*p* < 0.05 vs MGUS; ^*p* < 0.05 vs SWM.

In fact, MGUS cases showed decreased T-cell counts in blood (*p* = 0.03), particularly at the expense of TCD4^+^ naive cells (*p* = 0.02), Treg-like follicular helper (TFH) cells (*p* = 0.006), Tregs (*p* = 0.002)—and their naive, Th1-, Th2- and Th17-like subsets (*p* = ≤0.007)–, Th2 (*p* = 0.008)—and Th2-effector memory cells (*p* < 0.001)—and Th1-17 cells (*p* = 0.001)—Th1-Th17-central, transitional and effector memory cells (*p* ≤ 0.003)—together with increased numbers of blood-circulating TFH-naive cells (*p* < 0.001). In turn, decreased TCD4^+^ naive cells (*p* = 0.04) and Treg-naive, Th1-like, Th2-like and Th17-like Tregs (*p* ≤ 0.02), together with increased TFH-naive (*p* = 0.006) and Th22 cells (*p* = 0.004)—Th22-central and effector memory cells (*p* ≤ 0.04)—were found in SMM, whereas Treg-like (*p* = 0.05) and Th1-17-like (*p* = 0.05) TFH-cells, together with Tregs (*p* = 0.03)—and their Th1-, Th17- and Th22-like subsets (*p* ≤ 0.05)– were those TCD4^+^ subsets altered in SWM. Overall, these findings suggest a reduced TCD4^+^ cell and Treg production, with an increased recruitment of specific functional compartments of these latter cells to the tumor tissue (e.g., BM) already at the earliest stages of MG, in line with previous observations in overt disease [3, 9, 10]. Despite this, only slightly (non-significantly) elevated percentages of TCD4^+^ cells were found in BM of MGUS and SMM. However, more detailed analysis of the distribution of the functional subsets of TCD4^+^ cells suggests a pro-inflammatory response, with increased memory and/or effector Th2 and Th1-17 cells in MGUS, together with decreased numbers of innate lymphoid cells (ILC) (*p* < 0.001) in blood, at the expense of their Th2- and Th17-inducing ILC2 (*p* = 0.01) and ILC3 (*p* < 0.001) subsets, respectively (Figs. 1–2 and S1; Tables S2–3) [11]. Interestingly, BM infiltration by immunosuppressive Tregs reported in SMM and SWM [2] appears to occur already in MGUS cases, which may trigger local immunosuppression already at an early stage, prior to disease progression.

**Fig. 2:**
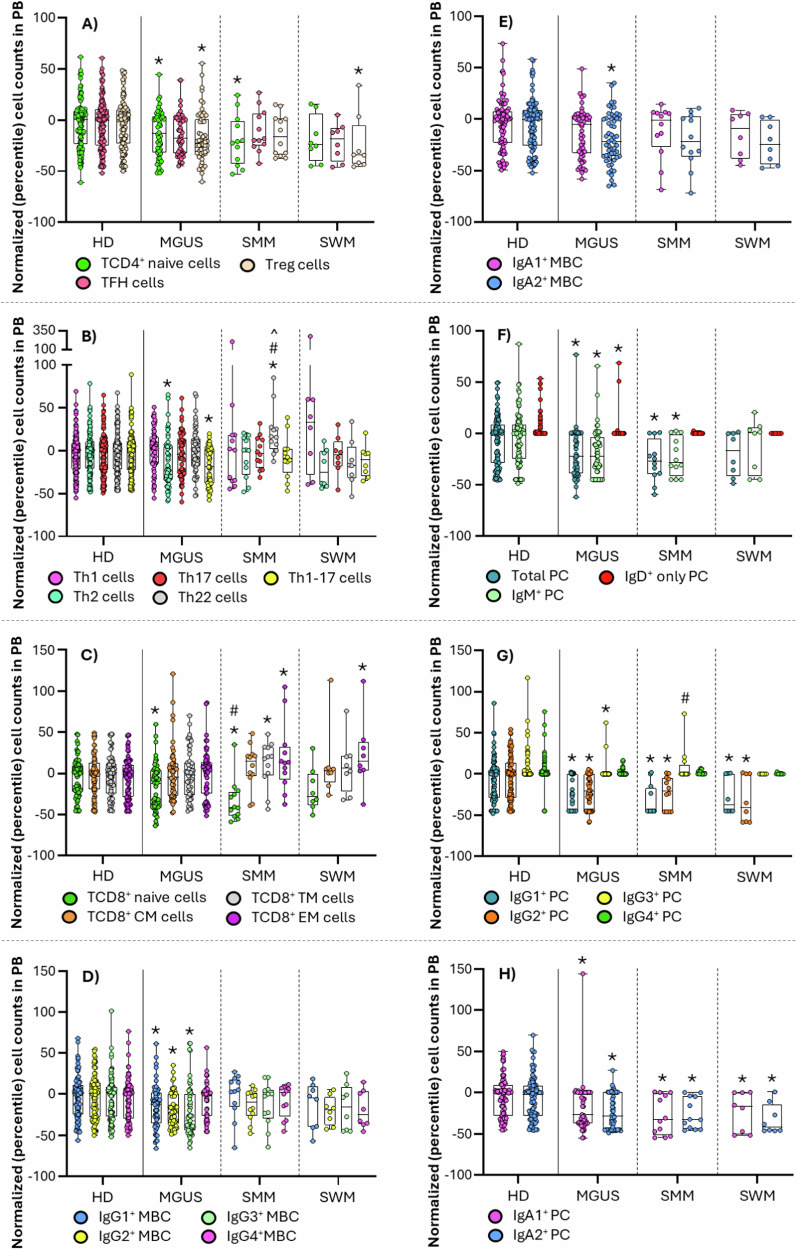
Distribution of the major T-cell, B-cell and plasma cell populations in blood of monoclonal gammopathy of undetermined significance (MGUS) (*n* = 55), smoldering multiple myeloma (SMM) (*n* = 12) and smoldering Waldenström’s macroglobulinemia (SWM) (*n* = 8) patients compared to healthy donors (HD) (*n* = 118). **A** TCD4^+^ naive, TFH and T-regulatory (Treg) cells; **B** Th1, Th2, Th17, Th22 and Th1-17 TCD4^+^ cells; **C** TCD8^+^ naive, central memory, transitional memory and effector memory cells; **D** IgG1^+^, IgG2^+^, IgG3^+^ and IgG4^+^ MBC; **E** IgA1^+^ and IgA2^+^ MBC; **F** total plasma cells (PC), IgM^+^ and IgD^+^ PC; **G** IgG1^+^, IgG2^+^, IgG3^+^ and IgG4^+^ PC; and **H** IgA1^+^ and IgA2^+^ PC. Box plots extend from the 25th to the 75th percentile values, while the horizontal lines indicate median values and both the minimum and maximum values (whiskers) of cell counts in blood normalized for each individual case (inner circles) by the median percentile values of aged-matched HD. HD, healthy donor; MBC, memory B-cells; MGUS, monoclonal gammopathy of undetermined significance; PC, plasma cells; preGC, pre-germinal center; SMM, smoldering multiple myeloma; SWM, smoldering Waldenström’s macroglobulinemia; TFH, follicular helper T-cell; Th, helper T-cell; Treg, regulatory T-cell. *p < 0.05 vs HD; #*p* < 0.05 vs MGUS; ^*p* < 0.05 vs SWM.

In parallel, TCD8^+^ naive cells, total NK-cells and their major CD56^lo^ cytotoxic NK-cell compartment [12] were all decreased in blood of MGUS (*p* ≤ 0.007) and SMM (*p* ≤ 0.01), while in WM, only the NK-cell populations were altered (*p* = 0.03). Likewise, the cytokine-secreting immunomodulatory [12] CD56^hi^ (cyGranzyme-B^+^ and CD57^-^) NK-cell population was also decreased in blood of MGUS (*p* = 0.03), while increased in BM of MGUS (*p* = 0.006) and SMM (*p* = 0.001) (but not SWM), suggesting an enhanced recruitment of progressively higher numbers of effector cytotoxic (e.g., TCD8^+^ and NK) cells to the tumor niche [1, 2, 9, 13]. Altogether, these findings point to an enhanced cytokine-secreting and immune-regulatory vs cytotoxic response at the earliest (MGUS) vs more advanced (e.g., SMM) disease stages, that might prevent excessive T-cell responses to proinflammatory stimuli already in MGUS [12, 14]. Of note, TCD8^+^ effector memory cells increased in SMM (*p* = 0.04) and SWM (*p* = 0.03), mostly showed a cyGranzyme-B^-^ CD57^-/+^ exhausted/immunosenescent phenotype, which might result from chronic exposure to target antigens in the cPC niche [9, 13]. Likewise, abnormally high TCD8^+^ cell numbers were found in BM of MGUS (*p* < 0.001) and SMM (*p* < 0.001), but not SWM. Globally, these data confirm previous observations pointing to the association between the emergence of oligoclonal expansions of cytotoxic T-cells in blood and in the tumor BM environment (as an attempt to control the outgrowth of cPC) in patients with MG [9, 13], and a better prognosis [9, 13]. In contrast, decreased Tγδ^+^ cytotoxic cell counts were identified in blood of SMM (*p* = 0.05) and in both blood (*p* = 0.02) and BM (*p* = 0.04 for CD3^hi^ Tγδ^+^ cells) of SWM, pointing towards a limited role of Tγδ^+^ cells in BM tumor surveillance at the earliest MG stages (Fig. 2 and S1; Tables S2-5).

Among all immune cells, B-cells were the most strikingly altered compartment. Thus, decreased counts of both total (normal residual) PC and B-lymphocytes—at the expense of pre-germinal center (GC) B-cells and memory B-cells (MBC)–, were found in blood of MGUS (*p* < 0.001) and SMM (*p* ≤ 0.01), while only MBC (*p* = 0.01) were decreased in SWM. Of note, unswitched-IgMD^+^ MBC (*p* ≤ 0.004) were decreased in blood across all patient groups, together with lower numbers of different subsets of switched-MBC in MGUS—IgG_1-3_ and IgA2^+^ (*p* ≤ 0.02)–, but not SMM and SWM. Interestingly, except for an increased percentage of precursor B-cells (*p* = 0.03) in MGUS, normal B-cell percentages were found in BM of all patient groups, suggesting an impaired generation of both switched-MBC and unswitched-MBC in MGUS, potentially due to a progressively decreased production and/or survival of (the more mature CD5^−^) naive B-lymphocytes. In SWM, however, the altered B-cell profile points to an intrinsically altered production and/or expansion of unswitched MBC, similar to SMM. Altogether, these results might reflect the generation of increased percentages of MBC is from pre-existing switched-MBC, due to a progressively more limited response to new antigens, and an increased risk of infection in more advanced disease stages [15]. In this regard, decreased numbers of recently-produced normal PC were observed in blood of MGUS (*p* < 0.001) and SMM (*p* = 0.01), which might be responsible in the medium-to-long-term immunoparesis observed in high-risk SMM and MM [15]. Interestingly, decreased IgM^+^, IgG_1-2_ and IgA_1-2_ PC counts were found in blood of MGUS (*p* < 0.001) and SMM (*p* ≤ 0.02), together with decreased serum IgM in non-IgM-MGUS (*p* = 0.03) and SMM (*p* < 0.001), and decreased serum IgG in IgM-MGUS (*p* < 0.001) (Figure S2). Conversely, SWM cases displayed decreased PC numbers –i.e., IgG_1-2_ (*p* ≤ 0.03) and IgA_1-2_ (*p* ≤ 0.007) switched-PC– associated with decreased serum IgG (*p* = 0.04) (but not uninvolved IgM) antibody levels, supporting an Ig class-switch defect in SWM, rather than a decreased ability to respond to new antigens (Figs. 1–2 and S2; Tables S2–3). Globally, these findings suggest a progressively defective production of naive B-lymphocytes associated with a decreased generation of PC and production of (IgM) antibodies against new antigens in (non-IgM) MGUS and SMM, leading to progressively decreased IgM serum levels and a potentially greater susceptibility to infection [15]. In contrast, in SWM, a specific PC Ig-class switch defect would exist, with decreased IgG_1-2_ and IgA_1-2_ PC counts, and serum IgG titers (similarly to IgM-MGUS), associated with normal preGC, switched-MBC and total PC numbers. Whether the defective IgM vs IgG immune responses observed here at the very early non-IgM-MGUS (and SMM) vs IgM-MGUS (and SWM) stages are a predisposing condition or a consequence of the clonally expanded tumor B-cell/PCs, remains to be elucidated.

Overall, our results revealed uniquely altered immune profiles already at the earliest MGUS stages, which specifically involved the B-cell and PC compartments, among other adaptative and innate immune cells. Of note, these altered immune profiles partially overlapped, but differed significantly from those observed in SMM and, more prominently even, in SWM (Figs. S3–4).

## Supplementary information

Supplemental Material

## Data Availability

The datasets generated and/or analyzed during the current study are available from the corresponding author on reasonable request.
