# Supplementary material for: Deep immune cell profiling in blood and bone marrow of early stage monoclonal gammopathy: an iStopMM and ECRIN-M3 collaborative study
Source: Blood Cancer J. 2025 Mar 27;15(1):46. doi: 10.1038/s41408-025-01255-3 (PMC11950208; doi:10.1038/s41408-025-01255-3)
Supplement: Supplementary file 1 — Supplemental Material [file 41408_2025_1255_MOESM1_ESM.docx]

**Supplementary Data S1**

**Material and methods**

**Patients, controls and samples**. Participants with MG were selected from the iStopMM study, a national population-based screening study for MG in Iceland and randomized trial of follow-up strategies described in detail elsewhere (1). Briefly, all residents of Iceland born in 1975 (n≈148,000) and before, were offered to participate, of whom 75,422 provided informed consent and underwent screening by capillary zone electrophoresis and free-light chain (FLC) assay (Binding Site, Birmingham, UK). Those with MGUS were randomized to three study arms with two of them being called in for active surveillance, some of them being diagnosed with SMM and SWM during that follow-up. For this study, 75 adults –41 males, 34 females; median (range) age: 67 (43-90 years)–, randomized to active clinical follow-up from MGUS in the iStopMM were selected by conveniency sampling. According to the International Myeloma Working Group (IMWG) criteria, 55 individuals were diagnosed with MGUS (18 IgM-MGUS; 37 non-IgM-MGUS), 12 had SMM and 8 SWM. From each individual, paired EDTA-anticoagulated blood (10 ml/sample; n=75) and BM-aspirated (2 ml/sample; n=75) samples were collected in Iceland and sent to the University of Salamanca (Salamanca, Spain) to be processed within 36 hours after collection. In parallel, 118 and 9 aged-matched HD were studied as controls for blood and BM analyses, respectively. For uninvolved serum Ig analyses, a group of 60 aged-matched HD were studied.

**Immunophenotypic identification, enumeration and characterization of leucocyte subsets in blood and BM**. Immunophenotypic identification, enumeration (i.e, cells/µl) and characterization of up to 360 (functional- and maturation-associated) populations of blood-circulating immune cells was performed on 4 mL of blood/subject, after staining for the EuroFlow 14-color/16-antibody innate myeloid immune monitoring cell panel (IMM-MoDC tube) and the 14-color/18-antibody EuroFlow IMM-IgH-isotype B-cell antibody combination (Table S1). For this purpose, the EuroFlow bulk-lyse standard operating procedures (SOP) for sample preparation and for staining of cell-surface and cell-surface plus intracellular markers (available at [www.euroflow.org](http://www.euroflow.org)) were used. In parallel, 200 µl of the same blood sample/patient was stained with the 13-color/13-antibody EuroFlow IMM-TCD4^+^ panel and the 12-color/12-antibody EuroFlow IMM-cytotoxic antibody combination (Table S1), using the EuroFlow SOP for staining of cell-surface and intracellular markers. Stained samples were acquired in LSRFortessa^TM^ (BD) or FACSymphony^TM^ (BD) flow cytometers for a median (range) of 2.5x10^6^ (1.1-5.9x10^6^), 4.3x10^6^ (1.2-10x10^6^), 0.8x10^6^ (0.09-4x10^6^) and 0.6x10^6^ (0.08-1.6x10^6^) cells for the EuroFlow-MoDC, BIgH, TCD4^+^ and cytotoxic IMM panels, respectively (Table S1). In parallel, paired BM (100 µl) and blood (100 µl) samples from a subset of 36 MGUS, 9 SMM and 3 SWM and 9 HD were stained with the EuroFlow-Lymphocyte-Screening Tube (LST), following the EuroFlow SOP; LST-stained cells were measured in a FACSLyric^TM^ (BD) flow cytometer for a median (range) of 1.3x10^6^ (0.2-5x10^6^) cells/sample. For data analysis, the Infinicyt^TM^ software (version 2.0.5.d; Cytognos SL., Salamanca, Spain) was used. For every sample, the limit of detection (LOD) of each cell population contained in it was set at ≥20 cells.

**Uninvolved serum immunoglobulin measurements.** Uninvolved serum IgM, IgG and IgA levels were measured following either the Heavylite® assay in an Optilite® turbidimeter (Binding Site) or conventional nephelometry (Dimension Vista; Siemens Healthcare, Erlanger, Germany).

**Statistical methods**. Median values and ranges were calculated for all continuous variables. The 5^th^-95^th^ percentile values from the 118 HD were used to calculate age-matched normalized values per patient (2) for all 360 immune cell subsets identified (Table S2) and for uninvolved serum IgM, IgG and IgA levels, where median values and the 5^th^-95^th^ percentiles equaled 0%, -45% and +45%, respectively. The Mann-Whitney U or the Kruskal-Wallis tests were used to assess the (two-sided) statistical significance of differences observed for continuous variables between ≥2 groups, respectively. For the identification of those immune cell populations that (most) contributed to discriminate HD from MGUS, SMM, and/or SWM cases, multivariate canonical analysis (CA) was used. Graphical representations including volcano plots representing those immune cell populations that displayed an altered distribution in MGUS, SMM and SWM (vs HD) were plotted with GraphPad Prism (version 8.0.2, GraphPad Software, Boston, MA) and MIDAS (version 2.0.5.d, Cytognos). For all other statistical analyses, the SPSS IBM-Statistical software Package for Social Sciences (SPSS v28.0; IBM Corp, Armonk, NY) was used. Statistical significance was set at *p*-values<0.05.

**References**

1. Rögnvaldsson S, Love TJ, Thorsteinsdottir S, Reed ER, Óskarsson JÞ, Pétursdóttir Í, et al. Iceland screens, treats, or prevents multiple myeloma (iStopMM): a population-based screening study for monoclonal gammopathy of undetermined significance and randomized controlled trial of follow-up strategies. Blood Cancer J. 2021;11(5):94.

2. Oliva-Ariza G, Fuentes-Herrero B, Carbonell C, Lecrevisse Q, Pérez-Pons A, Torres-Valle A, et al. High frequency of low-count monoclonal B-cell lymphocytosis in hospitalized COVID-19 patients. Blood. 2023;141(3):309–14.

**Supplementary Figure legends:**

**Supplementary Figure S1. Immune cell populations present in blood of monoclonal gammopathy of undetermined significance (MGUS) (n=55), smoldering multiple myeloma (SMM) (n=12) and smoldering Waldenström’s macroglobulinemia (SWM) (n=8) patients at significantly different levels than those found in age-matched healthy donors (HD) (n=118).** In all volcano plots, significance is set at p-values of <0.05 (log10 p-values 1.3) represented in the vertical Y-axis with fold changes of >1 and <-1 (twofold increased and decreased values, respectively) in the horizontal X-axis. Immune cell populations altered in blood of MGUS, SMM and SWM (vs HD) are shown as circles, squares and triangles, respectively, while once MGUS+SMM were considered together (vs SWM) are depicted as rhombus. Distinct colors denote the nature of the changes observed in the distribution of each cell population: grey, no significant changes; red, increased; and blue, decreased values. A: myeloid-cell populations; B: Tγδ^+^, TCD8^+^ and NK-cytotoxic cell populations; C: TCD4^+^ cell populations; and D: B-cell and plasma cell populations. cMo, classical monocytes; cyGranz-B, cytoplasmic Granzyme-B; EE, early effector; EM, effector memory; HD, healthy donor; ILC, innate lymphoid cell; MBC, memory B-cells; mDC, myeloid dendritic cells; MGUS, monoclonal gammopathy of undetermined significance; ncMo, non-classical monocytes; NK, natural-killer cells; PC, plasma cells; pDC, plasmacytoid dendritic cells; SMM, smoldering multiple myeloma; SWM, smoldering Waldenström’s macroglobulinemia; TE, terminal effector; TFH, follicular helper T-cell; Th, helper T-cell; TM, transitional memory; Treg, regulatory T-cell.

**Supplementary Figure S2. Uninvolved serum immunoglobulin levels in** **monoclonal gammopathy of undetermined significance (MGUS) (n=55), smoldering multiple myeloma (SMM) (n=12) and smoldering Waldenström’s macroglobulinemia (SWM) (n=8) patients compared to healthy donors (HD) (n=60).** Box plots extend from the 25th to the 75th percentile values, while the horizontal lines indicate median values and both the minimum and maximum values (whiskers) of cell counts in blood normalized for each individual case (inner circles) by the median percentile values of aged-matched HD. HD, healthy donor; MGUS, monoclonal gammopathy of undetermined significance; SMM, smoldering multiple myeloma; SWM, smoldering Waldenström’s macroglobulinemia. *p<0.05 vs HD; ^#^p<0.05 vs MGUS; ^p<0.05 vs SWM.

**Supplementary Figure S3. Multivariate canonical analysis based on the different immune cell populations present in blood of monoclonal gammopathy of undetermined significance (MGUS) (n=55), smoldering multiple myeloma (SMM) (n=12) and smoldering Waldenström’s macroglobulinemia (SWM) (n=8) patients compared to aged-matched healthy donors (HD).** Two-dimensional graphical representations of multivariate (canonical) analyses (CA) performed for each comparison are shown together with a table indicating the most informative cell populations in discriminating between the individual groups of patients and/or between them and HD, and their relative contribution (depicted as percentage values). Only those cell populations which showed statistically significant differences for each comparison in the univariate analysis (Mann-Whitney U test) were included in the multivariate CA. HD cases are depicted in green, MGUS are shown in blue, SMM in red and SWM in orange, while MGUS+SMM cases are displayed in pink. Round dots represent individual patients, while colored lines represent the corresponding second standard deviation (SD). Upward and downward arrows indicate increased and decreased values in the underlined diagnostic groups, respectively. Comparison between the immune profiles of HD vs MGUS are shown in A; HD vs SMM in B; HD vs SWM in C; HD vs MGUS vs SMM in D; MGUS vs SWM in E; and MGUS+SMM vs SWM in F. cMo, classical monocytes; HD, healthy donor; MBC, memory B-cells; MGUS, monoclonal gammopathy of undetermined significance; ncMo, non-classical monocytes; PC, plasma cells; SMM, smoldering multiple myeloma; SWM, smoldering Waldenström’s macroglobulinemia.

**Supplementary Figure S4. Altered immune cell profiles in blood of (IgM and non-IgM) MGUS, SMM and SWM patients.** Downward arrows and upward arrows indicate decreased and increased immune cell populations compared to HD, respectively. Cell populations showing normal cell counts are depicted in light blue, whereas altered cell numbers (vs HD) in MGUS are depicted in light yellow when decreased and dark yellow when increased, altered cells in SMM are displayed in light (decreased) and dark pink (increased) and in SWM in light (decreased) and dark orange (increased), respectively. BM, bone marrow; CM, central memory; DC, dendritic cell; EM, effector memory; HD, healthy donor; ILC, innate lymphoid cell; MBC; memory B-cell; mDC, myeloid dendritic cell; MGUS, monoclonal gammopathy of undetermined significance; M-MDSC; monocytic-myeloid-derived suppressor cells; NK, natural-killer; pDC, plasmacytoid dendritic cell; PMN, polymorphonuclear neutrophil; SMM, smoldering multiple myeloma; SWM, smoldering Waldenström’s macroglobulinemia; TFH, Follicular helper cell; Th, T helper cell; TM, transitional memory; Treg, regulatory T-cell.

**Supplementary Figure S1**

**
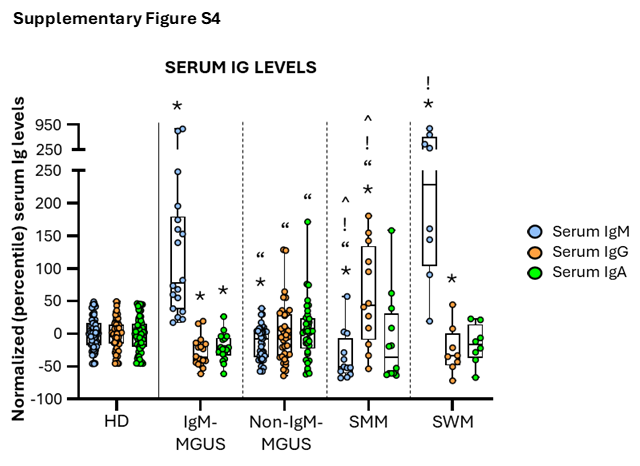
**

**Supplementary Figure S2**

**
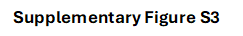

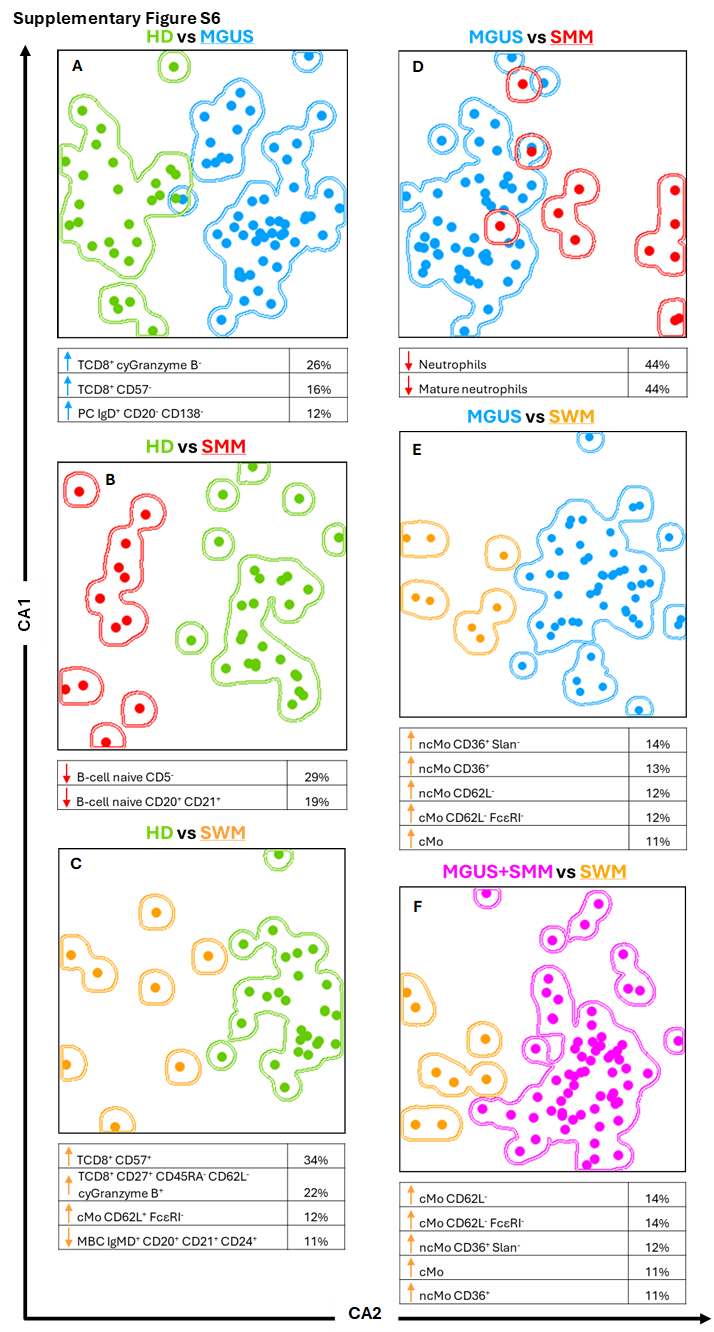
**

**
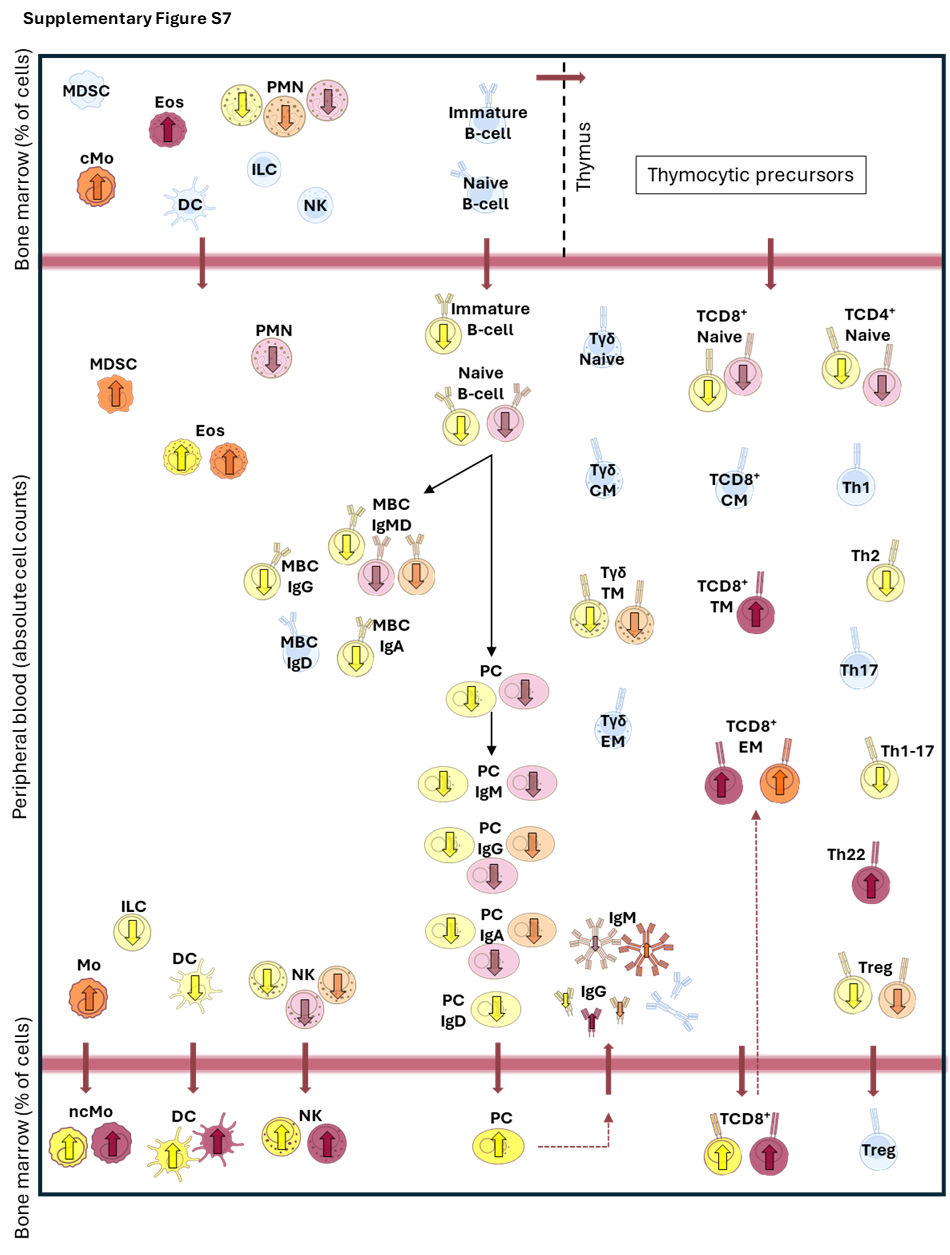

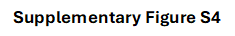
**

**Supplementary Table S1**. EuroFlow-Immune Monitoring (IMM) antibody panels used in this study for the immunophenotypic identification, enumeration and characterization of innate immune cells (A), TCD4^+^ cells (B), cytotoxic T/NK-cells (C) and both B-cells and plasma cells (D), in addition to the EuroFlow-Lymphocyte Screening Tube (LST; E).

| **Panel A** | **Fluorochrome** | **Marker** | | **Clone** | **Manufacturer** |
| --- | --- | --- | --- | --- | --- |
| **EuroFlow-Immune Monitoring (IMM) MoDC tube** | BV421 | CD141 | | 1A4 | BD |
|  | BV510 | CD5 | | UCHT2 | BD |
|  | BV605 | CD192 | | K036C2 | BioLegend |
|  | BV650 | CD62L | | DREG-56 | BioLegend |
|  | BV711 | HLA-DR | | G46-6 | BD |
|  | BV786 | CD16 | | 3G8 | BD |
|  | BB515 | CD1c | | F10/21A3 | BD Horizon |
|  | PerCP Cy5.5 | CD36 | | CLB-IVC7 | Immunostep |
|  | PE | Slan + FcεRI | | DD-1 + AER-37 | Miltenyi Biotech + Immunostep |
|  | PE CF594 | CD34 | | 581 | BD Horizon |
|  | PE Cy7 | CD33 | | P67.6 | BD |
|  | APC | CD300e + CD303 | | UP-H2 + AC144 | Immunostep + Miltenyi Biotech |
|  | AF 700 | CD45 | | HI30 | BD |
|  | APC H7 | CD14 | | MɸP9 | BD |
| **Panel B** | **Fluorochrome** | **Marker** | | **Clone** | **Manufacturer** |
| **EuroFlow-Immune Monitoring (IMM) TCD4+ tube** | BV421 | CD27 | | M-T271 | BD Horizon |
|  | BV510 | CD45RA | | HI100 | BD |
|  | BV650 | CD62L | | DREG-56 | BioLegend |
|  | BV711 | CD127 | | HIL7RM21 | BD |
|  | BV786 | CD3 | | SK7 | BD Horizon |
|  | VioBright FITC | CD25 | | 4E3 | Miltenyi Biotech |
|  | PerCP Cy5.5 | CCR10 | | 1B5 | BD |
|  | PE | CD183 | | 1C6/CD183 | BD |
|  | PE CF594 | CD196 | | 11A9 | BD |
|  | PE Cy7 | CD194 | | L291H4 | BioLegend |
|  | APC | CD185 | | REA103 | Miltenyi Biotech |
|  | AF 700 | CD45 | | HI30 | BD |
|  | APC H7 | CD4 | | SK3 | BD |
| **Panel C** | **Fluorochrome** | **Marker** | | **Clone** | **Manufacturer** |
| **EuroFlow-Immune Monitoring (IMM) Cytotoxic tube** | BV421 | CD27 | | M-T271 | BD Horizon |
|  | BV510 | CD45RA | | HI100 | BD |
|  | BV650 | CD62L | | DREG-56 | BioLegend |
|  | BV711 | CD16 | | 3G8 | BD |
|  | BV786 | CD3 | | SK7 | BD Horizon |
|  | FITC | CD57 | | HNK1 | BD |
|  | PerCP Cy5.5 | CD28 | | CD28.2 | BioLegend |
|  | PE | cyGranzyme-B | | GB-11 | Sanquin |
|  | PE CF594 | CD8 | | RPA-T8 | BD |
|  | PE Cy7 | TCRγδ | | 11F2 | BD |
|  | AF 700 | CD45 | | HI30 | BD |
|  | APC Vio770 | CD56 | | REA196 | Miltenyi Biotech |
| **Panel D** | **Fluorochrome** | **Marker** | | **Clone** | **Manufacturer** |
| **EuroFlow-IgH isotype tube** | BV421 | CD27 |  | M-T271 | BD Horizon |
|  | BV510 | IgM |  | MHM-88 | BioLegend |
|  | BV605 | CD62L |  | DREG-56 | BioLegend |
|  | BV650 | CD24 |  | ML5 | BD Horizon |
|  | BV711 | CD21 |  | Bly4 | BD |
|  | BV786 | CD19 |  | SJ25C1 | BD Horizon |
|  | FITC | smIgD | cyIgD | 1A6-2 | BioLegend |
|  | FITC | smIgG2 + smIgG3 | cyIgG2 + cyIgG3 |  | Cytognos (CYT-IGS-1) |
|  | PerCP Cy5.5 | smIgA1 + smIgA2 | cyIgA1 + cyIgA3 |  |  |
|  | PE | smIgG1 + smIgG2 | cyIgG1 + cyIgG3 |  |  |
|  | PE CF594 | CD20 |  | 2H7 | BD Horizon |
|  | PE Cy7 | CD5 + CD138 |  | L17F12 + MI15 | BD + BioLegend |
|  | APC | smIgD | cyIgD | 1A6-2 | BD |
|  | APC | smIgG4 + smIgA1 | cyIgG4 + cyIgA1 |  | Cytognos (CYT-IGS-1) |
|  | AF 700 | CD45 |  | HI30 | BD |
|  | APC H7 | CD38 |  | HB7 | BD |
| **Panel E** | **Fluorochrome** | **Marker** | | **Clone** | **Manufacturer** |
| **Euroflow- Lymphocyte Screening Tube (LST)** | HV450 | CD4 + CD20 | | SK3 + L279 | BD Biosciences OneFlow LST^TM^ |
|  | HV500c | CD45 | | 2D1 |  |
|  | FITC | CD8+ smIgλ | | SK1 + 1-155-2 |  |
|  | PE | CD56 + smIgκ | | MY31 + TB28-2 |  |
|  | PerCP Cy5.5 | CD5 | | L17F12 |  |
|  | PE Cy7 | CD19 + TCRγδ | | SJ25-C1 + 11F2 |  |
|  | APC | smCD3 | | SK7 |  |
|  | APC H7 | CD38 | | HB7 |  |

AF 700, alexa fluor 700; APC, allophycocyanine; APC H7, allophycocyanine-hilite®7; BB, brilliant blue; BD, Becton/Dickinson; BV, brilliant violet; cy, cytoplasmic; FITC; fluorescein isothiocyanate; HV, horizon violet dye; Ig, immunoglobulin; MoDC, monocyte cell and dendritic cell tube; PE, phycoerythrin; PE CF594, phycoerythrin cyanine-based 594 fluorescent dye; PE Cy7, phycoerythrin cyanine7; PerCP Cy5.5, peridinin chlorophyll protein cyanine5.5; sm, surface membrane; VioBright FITC; VioBright fluorescein isothiocyanate.

**Supplementary Table S2**. Distribution of the major and minor populations of innate myeloid cells (A), TCD4^+^ cells (B), cytotoxic T/NK-cells (C) and both B-cells and plasma cells (D) identified in blood of monoclonal gammopathy of undetermined significance (MGUS) (n=55) –IgM-MGUS (n=18) and non-IgM-MGUS (n= 37)–, smoldering multiple myeloma (SMM) (n=12) and smoldering Waldenström’s macroglobulinemia (SWM) (n=8) patients compared to aged-matched healthy adults (n=118).

| **A: Innate myeloid cells** | **Total MGUS** | **IgM-MGUS** | **Non-IgM-MGUS** | **SMM** | **SWM** | ***p* value** |
| --- | --- | --- | --- | --- | --- | --- |
| **Leucocytes** | 3 (-51 / 43) | 1 (-42 / 43) | 6 (-51 / 38) | -14^!^^ (-19 / 22) | 10" (-22 / 23) | *0.18* |
| **Eosinophils** | 6* (-63 / 259) | 18* (-47 / 57) | 5* (-63 / 259) | 1 (26 / 44) | 17* (-26 / 65) | *0.06* |
| **Neutrophils** | 6 (-88 / 48) | -4 (-82 / 34) | 10 (-88 / 48) | -23*^#!^ (-27 / 6) | 4 (-66 / 21) | *0.02* |
| Mature neutrophils | 6 (-88 / 48) | -4 (-82 / 34) | 10 (-88 / 48) | -23*^#!^ (-28 / 6) | 4 (-66 / 21) | *0.02* |
| Immature neutrophils | -3 (-51 / 527) | 1 (-51 / 527) | -11 (-46 / 69) | -13^ (-3 / 8) | 5 (-44 / 22) | *0.17* |
| Immature neutrophils CD16^-^CD62L^-^ | -5 (-51 / 392) | 1 (-51 / 392) | -12 (-47 / 56) | -13^ (-6 / 5) | 5 (-35 / 18) | *0.15* |
| Immature neutrophils CD16^-^CD62L^+^ | 2 (-42 / 313) | 5 (-40 / 313) | 1 (-42 / 68) | -11 (-1 / 34) | 3 (-41 / 53) | *0.54* |
| **Basophils** | -6 (-70 / 83) | -13 (-64 / 68) | -4 (-70 / 83) | -9 (9 / 33) | 6 (-69 / 34) | *0.86* |
| **Monocytes** | -3 (-55 / 74) | -10 (-44 / 74) | 4 (-55 / 60) | -16^ (5 / 41) | 39*^#^"^!^ (3 / 101) | *0.003* |
| **cMo** | 2 (-45 / 113) | -7 (-35 / 113) | 4 (-45 / 61) | -6^ (3 / 40) | 47*^#^"^!^ (10 / 96) | *0.004* |
| cMo CD62L^+^FcεRI^+^ | -12 (-44 / 47) | 1 (-43 / 47) | -24 (-44 / 42) | -11 (9 / 19) | 3 (-23 / 35) | *0.004* |
| cMo CD62L^+^FcεRI^-^ | 11* (-42 / 112) | 8 (-29 / 44) | 13* (-42 / 112) | 5 (34 / 34) | 19* (2 / 47) | *0.42* |
| cMo CD62L^-^FcεRI^+^ | -23* (-44 / 113) | -20 (-43 / 66) | -29* (-44 / 113) | -15^ (7 / 10) | 11*^#^"^!^ (1 / 35) | *0.003* |
| cMo CD62L^-^FcεRI^-^ | -21 (-49 / 76) | -22 (-43 / 76) | -19 (-49 / 49) | -26^ (3 / 25) | 21*^#^"^!^ (9 / 67) | *0.004* |
| cMo CD62L^+^ | 7* (-33 / 83) | 2 (-18 / 83) | 10* (-33 / 80) | 5 (28 / 55) | 31* (1 / 84) | *0.02* |
| cMo CD62L^-^ | -23* (-54 / 110) | -26 (-50 / 110) | -23 (-54 / 49) | -37*^ (2 / 25) | 25*^#^"^!^ (12 / 64) | *<0.001* |
| cMo FcεRI^+^ | -17 (-44 / 42) | -8 (-43 / 42) | -20 (-44 / 35) | -16^ (8 / 16) | 5^!^ (-18 / 34) | *0.24* |
| cMo FcεRI^-^ | 7 (-68 / 99) | 1 (-68 / 65) | 8 (-53 / 99) | -5^ (24 / 24) | 29*^#^"^!^ (5 / 85) | *0.009* |
| **iMo** | -1 (-49 / 51) | -3 (-49 / 25) | 0 (-43 / 51) | -17 (12 / 48) | 1 (-30 / 87) | *0.72* |
| **ncMo** | -21* (-58 / 353) | -23* (-58 / 34) | -14* (-49 / 353) | -19 (12 / 27) | 6^#^" (-32 / 136) | *0.004* |
| ncMo CD36^+^Slan^-^ | -19* (-50 / 37) | -30* (-50 / 9) | -16* (-47 / 37) | -22* (20 / 19) | 9^#^"^!^ (-34 / 44) | *0.002* |
| ncMo CD36^-^Slan^-^ | -5 (-47 / 682) | -2 (-47 / 41) | -7 (-41 / 682) | -22^ (16 / 34) | -1 (-15 / 49) | *0.28* |
| ncMo CD36^+^Slan^+^ | -34* (-52 / 1) | -32* (-46 / 1) | -35* (-52 / 0) | -34* (8 / 4) | -32* (-47 / 11) | *<0.001* |
| ncMo CD36^-^Slan^+^ | -16* (-50 / 77) | -15* (-46 / 25) | -18* (-50 / 77) | -14 (13 / 20) | -2 (-43 / 653) | *0.03* |
| ncMo CD36^+^ | -20* (-53 / 36) | -32* (-53 / 8) | -18* (-49 / 36) | -24* (17 / 18) | 7^#^"^!^ (-39 / 43) | *<0.001* |
| ncMo CD36^-^ | -11* (-60 / 414) | -17* (-60 / 36) | -9* (-54 / 414) | -17 (8 / 28) | 5 (-39 / 165) | *0.02* |
| ncMo Slan^+^ | -18* (-51 / 78) | -16* (-46 / 23) | -19* (-51 / 78) | -15 (13 / 19) | -3 (-44 / 612) | *0.005* |
| ncMo Slan^-^ | -17* (-48 / 429) | -20* (-48 / 31) | -17* (-42 / 429) | -23*^ (15 / 30) | 1^#^"^!^ (-24 / 44) | *0.006* |
| **Dendritic cells** | -20* (-59 / 55) | -30 (-59 / 52) | -19 (-55 / 55) | -23 (6 / 36) | -12 (-61 / 49) | *0.26* |
| **mDC** | -19* (-55 / 84) | -16 (-55 / 45) | -19 (-54 / 84) | -13 (11 / 24) | -2 (-54 / 25) | *0.27* |
| mDC CD1c^+^ | -18* (-54 / 86) | -15 (-54 / 42) | -19 (-51 / 86) | -13 (11 / 26) | -3 (-54 / 24) | *0.29* |
| mDC CD1c^+^CD14dim | 0 (-65 / 110) | 4 (-49 / 40) | -2 (-65 / 110) | -7 (19 / 79) | 7 (-22 / 34) | *0.80* |
| mDC CD1c^+^CD14^-^CD5^-^ | -22* (-60 / 94) | -29* (-53 / 50) | -20* (-60 / 94) | -19 (7 / 50) | -8 (-61 / 21) | *0.06* |
| mDC CD1c^+^CD14^-^CD5^+^ | -18* (-53 / 40) | -20* (-53 / 26) | -13 (-47 / 40) | -4 (1 / 47) | 1 (-46 / 9) | *0.09* |
| mDC CD141^+^ | -16* (-65 / 32) | -19* (-65 / 32) | -15* (-58 / 22) | -27* (4 / 10) | -13 (-64 / 26) | *0.007* |
| **pDC** | -14 (-77 / 83) | -24 (-77 / 73) | -13 (-61 / 83) | -20 (-13 / 37) | -48* (-75 / 80) | *0.08* |
| **Axl^+^ DC** | -16* (-51 / 34) | -20 (-50 / 34) | -16* (-51 / 25) | -23* (10 / 5) | -28* (-51 / 7) | *0.02* |
| **CD100^+^ DC precursors** | 3 (-59 / 37) | 4 (-19 / 16) | 2 (-59 / 37) | 3 (31 / 15) | -5 (-46 / 20) | *0.40* |
| **M-MDSC** | 3 (-35 / 58) | 5 (-11 / 42) | 3 (-35 / 58) | -7"^ (16 / 14) | 12*^#!^ (4 / 26) | *0.02* |
| **HPC** | -8 (-58 / 66) | -1 (-38 / 53) | -13 (-58 / 66) | -11 (-2 / 24) | 8 (-20 / 47) | *0.37* |
| **ILC** | -17* (-65 / 75) | -15 (-57 / 26) | -20* (-65 / 75) | -9 (-34 / 13) | -9 (-29 / 47) | *0.003* |
| ILC2 | -45* (-45 / 53) | -28 (-45 / 31) | -45* (-45 / 53) | -25 (-45 / 42) | -45 (-45 / 158) | *0.14* |
| ILC3 | -15* (-51 / 46) | -7 (-45 / 22) | -17* (-51 / 46) | -5 (-45 / 24) | -3 (-45 / 28) | *0.004* |

Results expressed as median (range) normalized (vs HD) absolute cell count (cells/µl) percentile values, with median values and both the 5^th^ and 95^th^ percentiles of HD corresponding to 0, -45 and +45 values.

HD, healthy donor; HPC, hematopoietic progenitor cells; iMo, intermediate monocytes; mDC, myeloid dendritic cells; max, maximum; MGUS, monoclonal gammopathy of undetermined significance; min, minimum; M-MDSC, monocytic myeloid-derived suppressor dells; ncMo, non-classical monocytes; pDC, plasmacytoid dendritic cells; SMM, smoldering multiple myeloma; SWM, smoldering Waldenström’s macroglobulinemia. **p*<0.05 vs HD; ^#^*p*<0.05 vs MGUS; “*p*<0.05 vs IgM-MGUS; ^!^*p*<0.05 vs IgM-MGUS; ^*p*<0.05 vs SWM.

**Supplementary Table S2** (continued).

| **B: TCD4^+^ cells** | **Total MGUS** | **IgM-MGUS** | **Non-IgM-MGUS** | **SMM** | **SWM** | ***p* value** |
| --- | --- | --- | --- | --- | --- | --- |
| **Total T-cells** | -11* (-52 / 34) | -16 (-52 / 33) | -10 (-46 / 34) | 8 (-43 / 35) | 0 (-47 / 31) | *0.24* |
| **TCD4^+^ cells** | -16 (-54 / 69) | -15 (-54 / 69) | -17 (-45 / 39) | -7 (-49 / 47) | -6 (-51 / 36) | *0.47* |
| **TFH** | 1 (-53 / 72) | -1 (-53 / 72) | 2 (-40 / 53) | 3 (-31 / 44) | -23 (-44 / 42) | *0.54* |
| Treg | -18* (-45 / 39) | -12 (-42 / 39) | -20* (-45 / 39) | -19 (-42 / 27) | -18* (-46 / 5) | *0.04* |
| Naive | 11* (-24 / 245) | 9* (-22 / 245) | 12* (-24 / 120) | 15* (-12 / 73) | -2 (-41 / 85) | *<0.001* |
| Th1-like | -9 (-56 / 56) | -5 (-41 / 56) | -9 (-56 / 43) | 0 (-38 / 37) | -8 (-54 / 31) | *0.50* |
| Th2-like | 6 (-53 / 115) | -7 (-48 / 82) | 8 (-53 / 115) | -7 (-59 / 33) | 2 (-44 / 34) | *0.63* |
| Th17-like | 3 (-57 / 60) | 4 (-57 / 60) | 3 (-39 / 45) | -3 (-28 / 51) | -10 (-38 / 16) | *0.87* |
| Th1-Th17-like | -5 (-49 / 37) | 0 (-42 / 34) | -10 (-49 / 37) | 5 (-49 / 42) | -26* (-57 / 40) | *0.10* |
| CD183^+^CD194^+^CD196^-^CCR10^-^ | 2 (-50 / 99) | -6 (-48 / 99) | 4 (-50 / 47) | 4 (-31 / 28) | -21^#!^ (-59 / 12) | *0.30* |
| CD183^+^CD194^+^CD196^+^CCR10^-^ | 3 (-49 / 67) | 9 (-49 / 50) | 2 (-47 / 67) | 1^ (-23 / 69) | -21 (-43 / 29) | *0.37* |
| CD183^-^CD194^-^CD196^+^CCR10^-^ | -17* (-54 / 30) | -9 (-52 / 29) | -18* (-54 / 30) | 0^#!^ (-30 / 40) | -22 (-40 / 55) | *0.043* |
| CD183^-^CD194^-^CD196^-^CCR10^-^ | 2 (-45 / 67) | 1 (-45 / 17) | 2 (-31 / 67) | 2 (-45 / 32) | 6 (-19 / 58) | *0.256* |
| **Tregs** | -21* (-60 / 55) | -24* (-52 / 55) | -21* (-60 / 44) | -16 (-37 / 15) | -34* (-45 / 34) | *0.006* |
| Naive | -28* (-58 / 11) | -30* (-51 / 6) | -26* (-58 / 11) | -30* (-58 / 8) | -30 (-48 / 38) | *<0.001* |
| Th1-like | -28* (-80 / 12) | -31* (-80 / 6) | -28* (-62 / 12) | -31* (-46 / 14) | -40* (-66 / 22) | *<0.001* |
| Th2-like | -24* (-63 / 81) | -24 (-57 / 25) | -25* (-63 / 81) | -26* (-53 / 6) | -34 (-64 / 20) | *0.001* |
| Th17-like | -19* (-57 / 54) | -17 (-41 / 54) | -21* (-57 / 38) | -17* (-45 / 13) | -33* (-54 / 54) | *0.005* |
| Th22-like | 0 (-58 / 95) | 1 (-33 / 95) | -1 (-58 / 87) | 3^ (-27 / 87) | -25*^#^" (-53 / 2) | *0.13* |
| CXCR3^+^CCR4^+^CCR6^-^CCR10^+^ | 4 (-45 / 54) | 3 (-45 / 27) | 4 (-45 / 54) | 6^ (-16 / 75) | -13^#^" (-45 / 9) | *0.14* |
| CXCR3^+^CCR4^+^CCR6^-^CCR10^-^ | -3 (-54 / 84) | -7 (-41 / 39) | -2 (-54 / 84) | -9 (-41 / 32) | -31 (-71 / 26) | *0.24* |
| CXCR3^+^CCR4^+^CCR6^+^CCR10^-^ | -17 (-59 / 95) | -11 (-59 / 95) | -21 (-55 / 75) | -15 (-37 / 11) | -39*" (-53 / 38) | *0.07* |
| CXCR3^+^CCR4^+^CCR6^+^CCR10^+^ | -6 (-47 / 76) | -5 (-35 / 76) | -10 (-47 / 76) | 3 (-35 / 73) | -15 (-50 / 14) | *0.64* |
| CXCR3^-^CCR4^+^CCR6^-^CCR10^+^ | 1 (-45 / 51) | -5 (-45 / 51) | 7 (-45 / 43) | 11 (-60 / 67) | -16* (-60 / 25) | *0.19* |
| **Naive** | -13* (-52 / 45) | -5 (-52 / 45) | -16* (-52 / 23) | -22* (-53 / 24) | -24 (-45 / 16) | *0.04* |
| Total CM | -8 (-39 / 39) | -10 (-39 / 30) | -8 (-38 / 39) | -7 (-42 / 40) | -17 (-33 / 25) | *0.87* |
| Total TM | 0 (-52 / 38) | -3 (-49 / 38) | 1 (-52 / 37) | 2 (-49 / 66) | -18 (-42 / 42) | *0.74* |
| Total EM | 2 (-49 / 52) | -1 (-43 / 44) | 4 (-49 / 52) | 3 (-23 / 95) | -9 (-38 / 71) | *0.63* |
| Total TE | 3 (-45 / 94) | 3 (-45 / 94) | 2 (-45 / 71) | -22 (-45 / 296) | 0 (-45 / 515) | *0.74* |
| **Th1** | 3 (-55 / 51) | -5 (-55 / 51) | 4 (-47 / 40) | 2 (-44 / 208) | 33 (-39 / 277) | *0.28* |
| CM | -3 (-63 / 60) | -1 (-48 / 21) | -4 (-63 / 60) | -17 (-53 / 18) | -6 (-41 / 187) | *0.86* |
| TM | 0 (-62 / 47) | 0 (-53 / 47) | 1 (-62 / 34) | 3 (-60 / 60) | -1 (-48 / 35) | *0.80* |
| EM | 1 (-49 / 67) | 1 (-44 / 43) | 3 (-49 / 67) | 0 (-38 / 118) | -3 (-43 / 74) | *0.54* |
| TE | 3* (-45 / 95) | 3 (-45 / 95) | 4 (-45 / 76) | -25 (-45 / 316) | 5 (-45 / 539) | *0.35* |
| **Th2** | -23* (-58 / 66) | -23 (-58 / 31) | -21* (-57 / 66) | 1 (-48 / 21) | -25 (-44 / 12) | *0.06* |
| CM | -17 (-51 / 70) | -16 (-33 / 31) | -17 (-51 / 70) | -2 (-43 / 21) | -22 (-43 / 11) | *0.31* |
| TM | -8 (-49 / 39) | -13 (-49 / 39) | -7 (-49 / 18) | -12 (-57 / 60) | -5 (-55 / 49) | *0.47* |
| EM | -25* (-56 / 40) | -19* (-48 / 40) | -29* (-56 / 32) | -15 (-46 / 23) | -23 (-56 / 14) | *<0.001* |
| TE | 0 (0 / 37) | 0 (0 / 8) | 0 (0 / 37) | 0 (0 / 40) | 0 (0 / 14) | *0.60* |
| **Th17** | -4 (-60 / 62) | -1 (-60 / 48) | -4 (-42 / 62) | -3 (-31 / 32) | -5 (-46 / 31) | *0.99* |
| CM | -8 (-41 / 57) | -2 (-35 / 38) | -9 (-41 / 57) | -10 (-32 / 27) | -7 (-37 / 17) | *0.98* |
| TM | -1 (-50 / 56) | -5 (-47 / 41) | 1 (-50 / 56) | 1 (-20 / 43) | -1 (-22 / 53) | *0.81* |
| EM | 1 (-52 / 127) | 9 (-52 / 127) | 1 (-47 / 68) | 3 (-12 / 77) | -3 (-23 / 130) | *0.34* |
| TE | 0* (0 / 0) | 0 (0 / 0) | 0 (0 / 0) | 0 (0 / 0) | 0 (0 / 0) | *0.21* |
| **Th22** | 1 (-53 / 67) | 2 (-53 / 67) | 0 (-52 / 64) | 16*^#!^^ (-12 / 85) | -17 (-53 / 34) | *0.03* |
| CM | 2 (-50 / 74) | 8 (-33 / 37) | 1 (-50 / 74) | 14*^ (-14 / 51) | -15 (-46 / 8) | *0.12* |
| TM | -10 (-75 / 75) | -11 (-55 / 44) | -9 (-75 / 75) | 10^#^^ (-29 / 88) | -21 (-52 / 36) | *0.17* |
| EM | 4 (-47 / 114) | 7 (-39 / 114) | 1 (-47 / 40) | 25*^#!^^ (-17 / 93) | -8 (-53 / 69) | *0.003* |
| TE | 0 (0 / 48) | 0 (0 / 48) | 0 (0 / 33) | 0 (0 / 90) | 0 (0 / 0) | *0.31* |
| **Th1-17** | -18* (-57 / 20) | -14 (-57 / 11) | -20* (-52 / 20) | -9 (-47 / 39) | -10 (-35 / 21) | *0.02* |
| CM | -21* (-59 / 25) | -20* (-59 / 8) | -22* (-49 / 25) | -18 (-47 / 44) | -20 (-41 / 30) | *0.01* |
| TM | -19* (-50 / 23) | -18 (-50 / 13) | -21* (-50 / 23) | -5 (-51 / 35) | -9 (-51 / 26) | *0.06* |
| EM | -18* (-61 / 17) | -14 (-54 / 7) | -25* (-61 / 17) | -2 (-40 / 79) | -1 (-30 / 4) | *0.02* |
| TE | 0 (0 / 44) | 0 (0 / 14) | 0 (0 / 44) | 0 (0 / 39) | 0 (0 / 0) | *0.19* |
| **CXCR3^+^CCR4^+^CCR6^+^CCR10^+^** | 2 (-40 / 45) | 2 (-40 / 43) | 2 (-38 / 45) | 12*^ (-21 / 31) | -11 (-38 / 12) | *0.17* |
| CM | 1 (-46 / 52) | 0 (-45 / 42) | 2 (-46 / 52) | 6 (-30 / 24) | -11 (-47 / 11) | *0.40* |
| TM | 1 (-50 / 79) | -4 (-46 / 54) | 2 (-50 / 79) | 10*^ (-18 / 34) | -11 (-36 / 14) | *0.10* |
| EM | 3* (-45 / 64) | 5 (-45 / 45) | 3 (-45 / 64) | 13* (-19 / 40) | -6 (-28 / 27) | *0.05* |
| TE | 0 (0 / 122) | 0 (0 / 23) | 0 (0 / 122) | 0 (0 / 281) | 0 (0 / 0) | *0.37* |
| **CXCR3^+^CCR4^+^CCR6^+^CCR10^-^** | 2 (-53 / 46) | 2 (-53 / 38) | 2 (-52 / 46) | -6 (-30 / 48) | -6 (-40 / 23) | *0.94* |
| CM | 2 (-48 / 41) | -2 (-48 / 31) | 3 (-41 / 41) | -9 (-41 / 45) | -6 (-29 / 17) | *0.93* |
| TM | -2 (-50 / 60) | 1 (-50 / 46) | -3 (-48 / 60) | 0 (-24 / 54) | -6 (-39 / 42) | *0.96* |
| EM | 3 (-47 / 73) | 5 (-43 / 73) | 1 (-47 / 30) | 7 (-20 / 19) | 2 (-28 / 9) | *0.30* |
| TE | 0* (0 / 9) | 0 (0 / 0) | 0 (0 / 9) | 0 (0 / 0) | 0 (0 / 0) | *0.13* |
| **CXCR3^+^CCR4^+^CCR6^-^CCR10^+^** | 7* (-36 / 79) | 12 (-36 / 63) | 6* (-32 / 79) | 13*^ (-17 / 94) | 4 (-39 / 12) | *0.01* |
| CM | 1 (-37 / 72) | 8 (-32 / 45) | 1 (-37 / 72) | 5 (-19 / 74) | -6 (-46 / 6) | *0.36* |
| TM | 8* (-45 / 98) | 8* (-45 / 85) | 9* (-45 / 98) | 9* (-7 / 103) | 9 (-45 / 27) | *<0.001* |
| EM | 7* (-45 / 73) | 10* (-19 / 56) | 7* (-45 / 73) | 19*^ (-9 / 110) | 6 (-45 / 32) | *<0.001* |
| TE | 0 (0 / 69) | 0 (0 / 0) | 0 (0 / 69) | 0 (0 / 53) | 0 (0 / 0) | *0.56* |
| **CXCR3^+^CCR4^+^CCR6^-^CCR10^-^** | -5 (-62 / 134) | -15 (-62 / 134) | -5 (-48 / 38) | -12 (-35 / 45) | -4 (-70 / 20) | *0.93* |
| CM | -2 (-50 / 114) | -4 (-41 / 114) | 1 (-50 / 26) | -8 (-37 / 45) | -6 (-53 / 6) | *0.79* |
| TM | 1 (-66 / 57) | -9 (-36 / 45) | 1 (-66 / 57) | 0 (-21 / 43) | -14 (-64 / 56) | *0.71* |
| EM | -1 (-49 / 64) | 4 (-46 / 40) | -6 (-49 / 64) | 7* (-8 / 60) | -3 (-55 / 58) | *0.17* |
| TE | -6* (-45 / 32) | 0 (-45 / 32) | -45* (-45 / 19) | -22 (-45 / 56) | -45* (-45 / 48) | *0.002* |
| **CXCR3^+^CCR4^-^CCR6^+^CCR10^+^** | 0 (-45 / 48) | 0 (-45 / 48) | 0 (-45 / 28) | 3 (-45 / 52) | 5 (-45 / 28) | *0.35* |
| CM | 0 (0 / 44) | 0 (0 / 34) | 0 (0 / 44) | 0 (0 / 57) | 0 (0 / 0) | *0.69* |
| TM | 0 (0 / 42) | 0 (0 / 42) | 0 (0 / 34) | 0 (0 / 56) | 0 (0 / 55) | *0.56* |
| EM | 0 (0 / 145) | 0 (0 / 145) | 0 (0 / 28) | 0 (0 / 137) | 0 (0 / 0) | *0.25* |
| TE | 0 (0 / 0) | 0 (0 / 0) | 0 (0 / 0) | 0 (0 / 0) | 0 (0 / 0) | *0.53* |
| **CXCR3^+^CCR4^-^CCR6^-^CCR10^+^** | 1 (-45 / 173) | 0 (-45 / 76) | 1 (-45 / 173) | 28* (-45 / 78) | 12 (-45 / 44) | *0.19* |
| CM | 0 (0 / 155) | 0 (0 / 48) | 0 (0 / 155) | 22*^#!^ (0 / 67) | 6 (0 / 18) | *0.11* |
| TM | 0 (0 / 178) | 0* (0 / 0) | 0 (0 / 178) | 8*^#^"^!^ (0 / 176) | 0 (0 / 52) | *0.01* |
| EM | 0 (0 / 655) | 0 (0 / 617) | 0 (0 / 655) | 0 (0 / 153) | 0 (0 / 189) | *0.40* |
| TE | 0 (0 / 0) | 0 (0 / 0) | 0 (0 / 0) | 0^#^ (0 / 2230) | 0 (0 / 0) | *0.44* |
| **CXCR3^-^CCR4^-^CCR6^+^CCR10^-^** | -17 (-53 / 69) | -9 (-53 / 69) | -21* (-41 / 28) | -16 (-34 / 76) | -18 (-38 / 47) | *0.34* |
| CM | -12 (-52 / 40) | -2 (-47 / 40) | -13 (-52 / 33) | -10 (-30 / 40) | -14 (-36 / 37) | *0.86* |
| TM | -24* (-45 / 39) | -24 (-45 / 39) | -25* (-45 / 19) | -8 (-45 / 70) | -2 (-45 / 58) | *0.11* |
| EM | 0 (-45 / 47) | 0 (-45 / 47) | 0 (-45 / 37) | 0^!^ (0 / 113) | 7^#^ (0 / 36) | *0.15* |
| TE | 0 (0 / 0) | 0 (0 / 0) | 0 (0 / 0) | 0 (0 / 0) | 0 (0 / 0) | *0.27* |
| **CXCR3^-^CCR4^+^CCR6^-^CCR10^+^** | 2* (-64 / 122) | 1 (-52 / 57) | 3* (-64 / 122) | 7*^ (-21 / 216) | -18^#!^ (-43 / 17) | *0.02* |
| CM | 2 (-51 / 152) | 0 (-40 / 92) | 3 (-51 / 152) | 8*^ (-24 / 209) | -21^#!^ (-49 / 26) | *0.01* |
| TM | 8* (0 / 109) | 0 (0 / 32) | 10* (0 / 109) | 3^ (-45 / 306) | 0^#^"^!^ (-45 / 17) | *<0.001* |
| EM | 3 (-45 / 121) | 3 (-45 / 49) | 3 (-45 / 121) | 3 (-45 / 54) | -22 (-45 / 21) | *0.29* |
| TE | 0* (0 / 0) | 0 (0 / 0) | 0* (0 / 0) | 0^#^ (0 / 98) | 0 (0 / 0) | *0.06* |
| **CXCR3^-^CCR4^-^CCR6^-^CCR10^-^CXCR5^-^** | 0 (-48 / 52) | 0 (-45 / 9) | 0 (-48 / 52) | 0 (-32 / 16) | 1 (-32 / 3) | *0.74* |

Results expressed as median (range) normalized (vs HD) absolute cell count (cells/µl) percentile values, with median values and both the 5^th^ and 95^th^ percentiles of HD corresponding to 0, -45 and +45 values.

CM, central memory; EM, effector memory; HD, healthy donor; max, maximum; ILC, innate lymphoid cells; MGUS, monoclonal gammopathy of undetermined significance; min, minimum; SMM, smoldering multiple myeloma; SWM, smoldering Waldenström’s macroglobulinemia; TE, terminal effector; TFH, follicular helper T-cells; Th, helper T-cells; TM, transitional memory; Treg, regulatory T-cells. **p*<0.05 vs HD; ^#^*p*<0.05 vs MGUS; “*p*<0.05 vs IgM-MGUS; ^!^*p*<0.05 vs IgM-MGUS; ^*p*<0.05 vs SWM.

**Supplementary Table S2** (continued).

| **C: Cytotoxic T/NK-cells** | **Total MGUS** | **IgM-MGUS** | **Non-IgM-MGUS** | | **SMM** | **SWM** | | ***p* value** |
| --- | --- | --- | --- | --- | --- | --- | --- | --- |
| **Tγδ^+^ cells** | -4 (-54 / 19) | -5 (-51 / 16) | -4 (-54 / 19) | | -12* (-50 / 1) | -30*^#!^ (-51 / -1) | | *0.08* |
| Total Tγδ^+^ cyGranzB^+^ | 0 (-47 / 31) | 1 (-46 / 30) | 0 (-47 / 31) | | -15 (-40 / 5) | -17 (-45 / 8) | | *0.41* |
| Total Tγδ^+^ cyGranzB^-^ | -21* (-58 / 81) | -21 (-58 / 81) | -20* (-51 / 47) | | -24 (-42 / 19) | -38* (-51 / 5) | | *0.02* |
| Total Tγδ^+^ CD57^+^ | 0 (-46 / 40) | 1 (-45 / 35) | 0 (-46 / 40) | | -8 (-44 / 4) | -26 (-43 / 9) | | *0.25* |
| Total Tγδ^+^ CD57^-^ | -12 (-59 / 36) | -24 (-59 / 23) | -10 (-47 / 36) | | -18 (-36 / 4) | -25 (-49 / 12) | | *0.10* |
| **Naive** | 0 (-45 / 35) | 0 (-45 / 35) | 0 (-45 / 17) | | 0 (-45 / 15) | 0 (-45 / 0) | | *0.76* |
| cyGranzB^-^CD57^-^ | 0 (-45 / 18) | 0 (-45 / 17) | 0 (-45 / 18) | | 0 (-45 / 14) | 0 (0 / 3) | | *0.73* |
| cyGranzB^-^CD57^+^ | 0 (0 / 0) | 0 (0 / 0) | 0 (0 / 0) | | 0 (0 / 0) | 0 (0 / 0) | | *0.73* |
| cyGranzB^+^CD57^-^ | 0 (0 / 0) | 0 (0 / 0) | 0 (0 / 0) | | 0 (0 / 0) | 0 (0 / 0) | | *0.91* |
| cyGranzB^+^CD57^+^ | 0 (0 / 0) | 0 (0 / 0) | 0 (0 / 0) | | 0 (0 / 0) | 0 (0 / 0) | | *0.91* |
| **CM** | -8 (-46 / 56) | -7 (-46 / 56) | -10 (-45 / 31) | | -19 (-45 / 26) | -22 (-45 / 12) | | *0.30* |
| cyGranzB^-^CD57^-^ | -9 (-46 / 57) | -8 (-46 / 57) | -10 (-45 / 31) | | -19 (-45 / 26) | -22 (-45 / 12) | | *0.30* |
| cyGranzB^-^CD57^+^ | 0 (0 / 92) | 0 (0 / 92) | 0 (0 / 57) | | 0 (0 / 0) | 0 (0 / 0) | | *0.38* |
| **TM** | -21* (-47 / 61) | -26 (-47 / 61) | -17 (-47 / 32) | | -17 (-45 / 39) | -35* (-45 / 12) | | *0.10* |
| cyGranzB^-^CD57^-^ | -19* (-48 / 70) | -28 (-47 / 70) | -16 (-48 / 31) | | -14 (-45 / 42) | -34* (-45 / 13) | | *0.11* |
| cyGranzB^-^CD57^+^ | 0 (0 / 32) | 0 (0 / 21) | 0 (0 / 32) | | 0 (0 / 18) | 0 (0 / 0) | | *0.71* |
| **EM** | -25 (-45 / 147) | -25 (-45 / 147) | -25 (-45 / 64) | | -8 (-45 / 4) | -26 (-45 / 67) | | *0.54* |
| cyGranzB^-^CD57^-^ | 0* (-45 / 26) | -5 (-45 / 12) | 0* (-45 / 26) | | 1 (-45 / 28) | -22 (-45 / 1) | | *0.10* |
| cyGranzB^-^CD57^+^ | 0 (0 / 309) | 0 (0 / 187) | 0 (0 / 309) | | 0 (0 / 74) | 0 (0 / 0) | | *0.60* |
| cyGranzB^+^CD57^-^ | 0 (-45 / 834) | 0 (0 / 76) | 0 (-45 / 834) | | 0 (0 / 19) | 0 (0 / 68) | | *0.97* |
| cyGranzB^+^CD57^+^ | -45 (-45 / 158) | -35 (-45 / 158) | -45 (-45 / 72) | | -45 (-45 / 5) | -45 (-45 / 62) | | *0.25* |
| **CD27^+^CD45RA^-^CD62L^+^cyGranzB^+^** | -15 (-45 / 126) | -26 (-45 / 77) | 1 (-45 / 126) | | 4 (-45 / 31) | -10 (-45 / 12) | | *0.53* |
| CD57^-^ | -12 (-45 / 100) | -24 (-45 / 100) | -12 (-45 / 87) | | 4 (-45 / 40) | -10 (-45 / 21) | | *0.56* |
| CD57^+^ | 0 (-45 / 199) | 0 (-45 / 48) | 0 (-45 / 199) | | 0 (0 / 27) | 0 (0 / 7) | | *0.62* |
| **CD27^+^CD45RA^-^CD62L^-^cyGranzB^+^** | -21 (-45 / 89) | -28 (-45 / 39) | -3 (-45 / 89) | | -12 (-45 / 16) | -36 (-45 / 7) | | *0.40* |
| CD57^-^ | -13 (-45 / 47) | -35 (-45 / 35) | -1 (-45 / 47) | | -26 (-45 / 12) | -45 (-45 / 9) | | *0.29* |
| CD57^+^ | 0 (-45 / 252) | 0 (-45 / 69) | 0 (-45 / 252) | | 0 (0 / 50) | 0 (0 / 10) | | *0.69* |
| **EE** | -6 (-46 / 73) | -7 (-46 / 63) | -6 (-45 / 73) | | 1 (-46 / 10) | -28 (-37 / 6) | | *0.49* |
| cyGranzB^-^CD57^-^ | 1 (-45 / 71) | 3 (-45 / 47) | 1 (-45 / 71) | | 6^ (-45 / 11) | -45^#^ (-45 / 22) | | *0.13* |
| cyGranzB^-^CD57^+^ | 0 (0 / 43) | 0 (0 / 19) | 0 (0 / 43) | | 0 (0 / 49) | 0 (0 / 0) | | *0.56* |
| cyGranzB^+^CD57^-^ | 1 (-45 / 69) | 0 (-45 / 69) | 1 (-45 / 43) | | 0 (-45 / 5) | -22 (-45 / 6) | | *0.32* |
| cyGranzB^+^CD57^+^ | 0 (-45 / 61) | 0 (-45 / 61) | 0 (-45 / 33) | | 2 (-45 / 19) | -45 (-45 / 4) | | *0.52* |
| **TE** | 1 (-49 / 47) | -2 (-44 / 16) | 1 (-49 / 47) | | 1 (-46 / 15) | -8 (-44 / 9) | | *0.81* |
| cyGranzB^-^CD57^-^ | 2* (0 / 168) | 2* (0 / 35) | 2 (0 / 168) | | 3* (0 / 17) | 1 (0 / 16) | | *0.07* |
| cyGranzB^-^CD57^+^ | 0* (0 / 196) | 0* (0 / 22) | 0 (0 / 196) | | 0 (0 / 26) | 0^#^ (0 / 0) | | *0.08* |
| cyGranzB^+^CD57^-^ | 1 (-45 / 29) | 1 (-45 / 25) | 1 (-45 / 29) | | 1 (-45 / 12) | 6 (-45 / 33) | | *0.69* |
| cyGranzB^+^CD57^+^ | 1 (-48 / 54) | -3 (-46 / 15) | 1 (-48 / 54) | | -5 (-44 / 13) | -14 (-41 / 1) | | *0.60* |
| **TCD8^+^ cells** | 0 (-59 / 37) | -1 (-58 / 30) | 3 (-59 / 37) | | 6 (-53 / 123) | 17 (-21 / 66) | | *0.28* |
| Total TCD8^+^ cyGranzB^+^ | 10* (-50 / 84) | 4 (-46 / 61) | 13* (-50 / 84) | | 13 (-40 / 125) | 33* (-24 / 104) | | *0.01* |
| Total TCD8^+^ cyGranzB^-^ | 10* (-20 / 68) | 10* (-20 / 68) | 10* (-17 / 40) | | 10* (-24 / 29) | 12* (-7 / 35) | | *0.002* |
| Total TCD8^+^ CD57^+^ | 9* (-46 / 82) | 6 (-39 / 53) | 10* (-46 / 82) | | 15 (-38 / 110) | 33*^#^" (-35 / 99) | | *0.005* |
| Total TCD8^+^ CD57^-^ | 11* (-27 / 67) | 10* (-27 / 67) | 11* (-23 / 37) | | 12* (-29 / 36) | 16* (-9 / 34) | | *0.001* |
| **Naive** | -12* (-63 / 60) | -9 (-63 / 60) | -16* (-62 / 45) | | -41*^#!^ (-59 / 35) | -28 (-50 / 30) | | *0.001* |
| cyGranzB^-^CD57^-^ | -15* (-63 / 60) | -10 (-63 / 60) | -15* (-62 / 43) | | -42*^#!^ (-59 / 35) | -28 (-51 / 30) | | *0.001* |
| cyGranzB^-^CD57^+^ | 0 (0 / 179) | 0 (0 / 0) | 0 (0 / 179) | | 0 (0 / 68) | 0 (0 / 0) | | *0.72* |
| cyGranzB^+^CD57^-^ | 0 (0 / 230) | 0 (0 / 55) | 0 (0 / 230) | | 0 (0 / 25) | 0 (0 / 216) | | *0.35* |
| cyGranzB^+^CD57^+^ | 0 (0 / 442) | 0 (0 / 32) | 0 (0 / 442) | | 0 (0 / 45) | 0 (0 / 69) | | *0.87* |
| **CM** | -4 (-48 / 121) | -3 (-47 / 86) | -4 (-48 / 121) | | 15 (-39 / 48) | 3 (-26 / 114) | | *0.60* |
| cyGranzB^-^CD57^-^ | -6 (-49 / 116) | -4 (-46 / 85) | -6 (-49 / 116) | | 12 (-39 / 46) | 3 (-26 / 113) | | *0.68* |
| cyGranzB^-^CD57^+^ | 5* (-45 / 52) | 5* (-21 / 52) | 5* (-45 / 43) | | 6* (-45 / 79) | 5 (2 / 37) | | *0.01* |
| **TM** | -2 (-46 / 70) | -6 (-46 / 70) | -1 (-43 / 60) | | 19* (-43 / 48) | 6 (-32 / 76) | | *0.31* |
| cyGranzB^-^CD57^-^ | -6 (-49 / 62) | -10 (-47 / 62) | -3 (-49 / 59) | | 15 (-44 / 48) | 7 (-36 / 61) | | *0.33* |
| cyGranzB^-^CD57^+^ | 4* (-45 / 132) | 3* (-30 / 104) | 4* (-45 / 132) | | 13*^#^ (-18 / 53) | 8* (-25 / 66) | | *0.001* |
| **EM** | 6 (-51 / 86) | 2 (-51 / 40) | 8 (-45 / 86) | | 13* (-38 / 105) | 15* (-38 / 112) | | *0.06* |
| cyGranzB^-^CD57^-^ | -6 (-62 / 86) | -15 (-54 / 56) | -4 (-62 / 86) | | 10 (-48 / 422) | 12*^#^"^!^ (-18 / 238) | | *0.07* |
| cyGranzB^-^CD57^+^ | 2 (-45 / 57) | 3 (-45 / 43) | 2 (-45 / 57) | | 7* (-45 / 155) | 9 (-45 / 78) | | *0.186* |
| cyGranzB^+^CD57^-^ | 1 (-45 / 129) | 1 (-45 / 83) | 2 (-45 / 129) | 22*"^#^ (-38 / 1609) | | | 6 (-24 / 325) | *0.03* |
| cyGranzB^+^CD57^+^ | 4 (-45 / 77) | 1 (-44 / 43) | 7 (-45 / 77) | | 12 (-44 / 59) | 14* (-41 / 83) | | *0.05* |
| **CD27^+^CD45RA^-^CD62L^+^cyGranzB^+^** | 1 (-43 / 118) | 1 (-43 / 117) | 0 (-34 / 118) | | 7* (-27 / 185) | 1 (-24 / 159) | | *0.19* |
| CD57^-^ | 0 (-53 / 91) | 1 (-44 / 65) | -2 (-53 / 91) | | 7*^#!^ (-31 / 480) | 0 (-36 / 124) | | *0.21* |
| CD57^+^ | 0 (-36 / 95) | -1 (-36 / 88) | 1 (-35 / 95) | | 4* (-17 / 60) | 1 (-14 / 57) | | *0.18* |
| **CD27^+^CD45RA^-^CD62L^-^cyGranzB^+^** | 0 (-45 / 144) | -2 (-45 / 97) | 1 (-43 / 144) | | 8* (-33 / 71) | 11*^#^"^!^ (-1 / 99) | | *0.02* |
| CD57^-^ | 1 (-49 / 240) | -1 (-49 / 62) | 1 (-44 / 240) | | 10*"^#^ (-27 / 98) | 8 (-22 / 112) | | *0.04* |
| CD57^+^ | 1 (-42 / 80) | 1 (-42 / 80) | 1 (-34 / 36) | | 5*^#!^ (-24 / 46) | 7* (-10 / 49) | | *0.003* |
| **EE** | 0 (-47 / 40) | -5 (-47 / 8) | 0 (-43 / 40) | | -2 (-37 / 8) | 0 (-32 / 5) | | *0.96* |
| cyGranzB^-^CD57^-^ | 2 (-38 / 69) | 3 (-38 / 69) | 2 (-35 / 41) | | 1 (-36 / 18) | 3 (-39 / 13) | | *0.49* |
| cyGranzB^-^CD57^+^ | 5* (0 / 167) | 7* (0 / 126) | 5* (0 / 167) | | 4* (0 / 98) | 0 (0 / 18) | | *0.02* |
| cyGranzB^+^CD57^-^ | -11 (-52 / 146) | -3 (-52 / 24) | -12 (-47 / 146) | | -4 (-45 / 12) | -3 (-35 / 43) | | *0.98* |
| cyGranzB^+^CD57^+^ | 0 (-50 / 51) | 1 (-50 / 33) | -1 (-48 / 51) | | 0 (-39 / 70) | 2* (0 / 9) | | *0.25* |
| **TE** | 2 (-47 / 107) | -1 (-44 / 107) | 4 (-47 / 61) | | 9 (-44 / 78) | 7 (-42 / 112) | | *0.56* |
| cyGranzB^-^CD57^-^ | 2* (-45 / 56) | 2 (-45 / 56) | 2* (-45 / 32) | | 2 (-30 / 36) | 2 (-45 / 8) | | *0.09* |
| cyGranzB^-^CD57^+^ | 2* (-45 / 102) | 1 (-45 / 21) | 2* (-45 / 102) | | 3* (0 / 18) | 3 (-45 / 7) | | *0.002* |
| cyGranzB^+^CD57^-^ | 4 (-48 / 102) | 3 (-45 / 102) | 4 (-48 / 102) | | 14 (-45 / 53) | 3 (-37 / 74) | | *0.46* |
| cyGranzB^+^CD57^+^ | 3 (-46 / 111) | -1 (-44 / 111) | 4 (-46 / 50) | | 6 (-42 / 60) | 7 (-41 / 99) | | *0.53* |
| **TCD8^-^Tγδ^-^ cells** | -10 (-46 / 60) | -10 (-43 / 60) | -10 (-46 / 32) | | 2 (-46 / 26) | -2 (-48 / 45) | | *0.82* |
| cyGranzB^-^CD57^-^ | -8 (-49 / 61) | -7 (-49 / 61) | -12 (-31 / 32) | | -7 (-39 / 19) | -10 (-28 / 11) | | *0.85* |
| cyGranzB^-^CD57^+^ | 15* (-45 / 90) | 18* (-42 / 90) | 13* (-45 / 79) | | 17* (-72 / 52) | 12* (1 / 82) | | *0.001* |
| cyGranzB^+^CD57^-^ | 1 (-70 / 77) | -11 (-45 / 77) | 3 (-70 / 71) | | -6 (-43 / 136) | 4 (-40 / 96) | | *0.57* |
| cyGranzB^+^CD57^+^ | 9* (-46 / 111) | 7 (-44 / 45) | 10* (-46 / 111) | | 13 (-40 / 231) | 15 (-44 / 252) | | *0.08* |
| **NK-cells** | -29* (-63 / 40) | -37* (-63 / 40) | -21* (-60 / 31) | | -21* (-54 / 12) | -25* (-60 / 18) | | *0.001* |
| Total NK cyGranzB^+^ | -26* (-68 / 41) | -40* (-68 / 41) | -22* (-64 / 33) | | -18* (-57 / 14) | -26* (-62 / 20) | | *0.002* |
| Total NK cyGranzB^-^ | -11 (-63 / 22) | -12 (-48 / 10) | -11 (-63 / 22) | | 1 (-29 / 18) | 1 (-70 / 4) | | *0.50* |
| Total NK CD57^+^ | -17 (-63 / 67) | -16 (-46 / 67) | -19 (-63 / 62) | | -10 (-34 / 23) | -8 (-37 / 19) | | *0.35* |
| Total NK CD57^-^ | -35* (-67 / 36) | -32* (-64 / 15) | -35* (-67 / 36) | | -29* (-56 / -1) | -36* (-62 / 17) | | *<0.001* |
| **CD56^hi^** | -13 (-74 / 95) | -8 (-71 / 22) | -14* (-74 / 95) | | 8 (-28 / 19) | -1 (-81 / 19) | | *0.19* |
| cyGranzB^-^CD57^-^ | -7 (-76 / 60) | 4 (-51 / 32) | -12 (-76 / 60) | | 9^!^ (-21 / 42) | 4 (-62 / 25) | | *0.23* |
| cyGranzB^-^CD57^+^ | 0 (0 / 0) | 0 (0 / 0) | 0 (0 / 0) | | 0 (0 / 0) | 0 (0 / 0) | | *0.74* |
| cyGranzB^+^CD57^-^ | -18* (-50 / 224) | -21 (-45 / 24) | -17 (-50 / 224) | | -9 (-45 / 36) | 1 (-45 / 4) | | *0.22* |
| cyGranzB^+^CD57^+^ | 0* (-45 / 92) | 0* (-45 / 23) | 0* (-45 / 92) | | 0 (-45 / 14) | 0 (-45 / 12) | | *0.03* |
| **CD56^lo^** | -30* (-64 / 39) | -39* (-64 / 39) | -23* (-59 / 33) | | -22* (-55 / 11) | -26* (-59 / 19) | | *0.001* |
| cyGranzB^-^CD57^-^ | -14 (-46 / 19) | -8 (-35 / 11) | -16 (-46 / 19) | | -1 (-37 / 19) | 0 (-30 / 4) | | *0.78* |
| cyGranzB^-^CD57^+^ | 0 (-45 / 30) | 0 (-45 / 4) | 0 (-45 / 30) | | 2 (-45 / 8) | 0 (-45 / 2) | | *0.73* |
| cyGranzB^+^CD57^-^ | -32* (-69 / 34) | -31* (-64 / 24) | -32* (-69 / 34) | | -26* (-58 / -4) | -35* (-59 / 21) | | *<0.001* |
| cyGranzB^+^CD57^+^ | -14 (-64 / 67) | -14 (-46 / 67) | -21 (-64 / 64) | | -10 (-34 / 24) | -5 (-36 / 20) | | *0.49* |

Results expressed as median (range) normalized (vs HD) absolute cell count (cells/µl) percentile values, with median values and both the 5^th^ and 95^th^ percentiles of HD corresponding to 0, -45 and +45 values.

CM, central memory; cy, cytoplasmic; EE, early effector; EM, effector memory; GranzB, Granzyme-B; HD, healthy donor; max, maximum; MGUS, monoclonal gammopathy of undetermined significance; min, minimum; SMM, smoldering multiple myeloma; SWM, smoldering Waldenström’s macroglobulinemia; TE, terminal effector; TM, transitional memory. **p*<0.05 vs HD; ^#^*p*<0.05 vs MGUS; “*p*<0.05 vs IgM-MGUS; ^!^*p*<0.05 vs IgM-MGUS; ^*p*<0.05 vs SWM.

**Supplementary Table S2** (continued).

| **D: B-cells and plasma cells** | **Total MGUS** | **IgM-MGUS** | **Non-IgM-MGUS** | **SMM** | **SWM** | ***p value*** |
| --- | --- | --- | --- | --- | --- | --- |
| **B-cells** | -32* (-69 / 48) | -37* (-67 / 25) | -30* (-69 / 48) | -30* (-69 / 1) | -26 (-55 / 39) | *<0.001* |
| **Pre-GC** | -26* (-74 / 83) | -28* (-74 / 25) | -25* (-72 / 83) | -28* (-74 / 7) | -6 (-51 / 82) | *<0.001* |
| **Immature** | -18* (-62 / 71) | -15 (-57 / 12) | -27* (-62 / 71) | -17 (-57 / 15) | 1 (-49 / 33) | *0.08* |
| **Naive** | -26* (-75 / 86) | -28* (-75 / 27) | -26* (-72 / 86) | -27* (-71 / 6) | -9 (-51 / 84) | *<0.001* |
| CD5^+^ | -18* (-50 / 97) | -11 (-49 / 18) | -18 (-50 / 97) | 5 (-50 / 14) | 6 (-44 / 35) | *0.15* |
| CD5^-^ | -30* (-74 / 103) | -33* (-74 / 35) | -28* (-67 / 103) | -34* (-74 / 3) | -17 (-53 / 109) | *<0.001* |
| CD20^+^CD21^+^ | -27* (-74 / 102) | -29* (-74 / 35) | -24* (-68 / 102) | -32* (-61 / 3) | -17 (-50 / 110) | *<0.001* |
| CD20^+^CD21^+^CD62L^+^ | -38* (-47 / 107) | -39* (-46 / 6) | -38* (-47 / 107) | -35* (-47 / 0) | -36 (-44 / 179) | *<0.001* |
| CD20^+^CD21^+^CD62L^-^ | -6 (-52 / 48) | -4 (-52 / 35) | -6 (-51 / 48) | -13 (-45 / 12) | 3 (-36 / 24) | *0.59* |
| CD20^++^CD21^-^ | -24* (-58 / 31) | -8 (-58 / 29) | -26* (-49 / 31) | -23* (-50 / 14) | -14 (-36 / 60) | *0.02* |
| **MBC** | -30* (-67 / 24) | -32* (-62 / 24) | -30* (-67 / 13) | -17* (-69 / 2) | -34* (-42 / 10) | *<0.001* |
| Total MBC CD27^+^ | -22* (-55 / 26) | -20 (-48 / 26) | -23* (-55 / 16) | -15 (-56 / 6) | -26 (-35 / 13) | *0.03* |
| Total MBC CD27^-^ | -33* (-49 / 7) | -40* (-49 / 7) | -29* (-46 / 3) | -26* (-51 / 11) | -23 (-41 / 0) | *<0.001* |
| Total MBC CD21^+^ | -29* (-60 / 27) | -28* (-54 / 27) | -32* (-60 / 11) | -18* (-62 / 1) | -38* (-41 / 7) | *<0.001* |
| Total MBC CD21^-^ | -35* (-64 / 27) | -37* (-64 / 7) | -30* (-59 / 27) | -16* (-62 / 9) | -30* (-50 / 32) | *<0.001* |
| **IgMD^+^** | -38* (-72 / 13) | -24* (-63 / 12) | -41* (-72 / 13) | -35* (-66 / -14) | -41* (-61 / 8) | *<0.001* |
| CD20^+^CD21^+^CD24^+^ | -36* (-70 / 14) | -19* (-57 / 14) | -39* (-70 / 10) | -35* (-60 / -11) | -40* (-57 / 7) | *<0.001* |
| CD20^++^CD21^-^ | -39* (-56 / 19) | -43* (-56 / 0) | -37* (-56 / 19) | -42* (-58 / -16) | -43* (-55 / 14) | *<0.001* |
| CD20^++^CD21^-^CD24^+^ | -34* (-53 / 23) | -37* (-51 / 1) | -32* (-53 / 23) | -33* (-43 / -7) | -32 (-43 / 11) | *<0.001* |
| CD20^++^CD21^-^CD24^-^ | -28* (-66 / 14) | -33* (-66 / 13) | -26* (-66 / 14) | -34* (-59 / 14) | -9 (-57 / 9) | *<0.001* |
| **IgG1^+^** | -13* (-66 / 61) | -26 (-57 / 61) | -12* (-66 / 37) | 4^!^ (-65 / 27) | -4 (-57 / 19) | *0.10* |
| CD20^+^CD21^+^CD24^+^ | -16* (-61 / 75) | -24 (-56 / 75) | -12* (-61 / 38) | 5 (-60 / 28) | 0 (-51 / 8) | *0.13* |
| CD20^+^CD21^+^CD24^+^CD27^+^ | -9 (-60 / 101) | -18 (-57 / 101) | -9 (-60 / 41) | 6 (-64 / 40) | -2 (-54 / 15) | *0.40* |
| CD20^+^CD21^+^CD24^+^CD27^-^ | -23* (-51 / 36) | -33* (-51 / 36) | -22* (-49 / 26) | -18 (-54 / 58) | -19 (-39 / 5) | *0.003* |
| CD20^++^CD21^-^ | -13* (-63 / 34) | -15* (-63 / 17) | -11* (-63 / 34) | 8^#^"^!^ (-60 / 34) | -12 (-51 / 57) | *0.02* |
| CD20^++^CD21^-^CD24^+^ | -22* (-59 / 19) | -28* (-59 / 11) | -21* (-51 / 19) | 0^#^"^!^ (-55 / 22) | -25 (-46 / 11) | *0.003* |
| CD20^++^CD21^-^CD24^+^CD27^+^ | -17* (-50 / 32) | -22* (-50 / 16) | -14 (-43 / 32) | -4" (-46 / 30) | -28 (-41 / 14) | *0.05* |
| CD20^++^CD21^-^CD24^+^CD27^-^ | -25* (-45 / 25) | -28* (-45 / 8) | -23* (-45 / 25) | 0^#^" (-45 / 57) | -20 (-25 / 2) | *0.003* |
| CD20^++^CD21^-^CD24^-^ | -18* (-63 / 89) | -24 (-63 / 89) | -15 (-60 / 83) | 13^#^"^!^ (-59 / 58) | -5 (-57 / 321) | *0.06* |
| CD20^++^CD21^-^CD24^-^CD27^+^ | -3 (-46 / 273) | -17 (-46 / 65) | 0 (-46 / 273) | 16*^#^"^!^ (-33 / 98) | -8 (-46 / 236) | *0.18* |
| CD20^++^CD21^-^CD24^-^CD27^-^ | -12* (-69 / 56) | -13 (-57 / 56) | -12 (-69 / 29) | 5^#^"^!^ (-40 / 41) | 3 (-58 / 173) | *0.04* |
| **IgG2^+^** | -22* (-49 / 35) | -30* (-49 / 35) | -21* (-47 / 27) | -10 (-48 / 10) | -19 (-43 / 5) | *0.005* |
| CD20^+^CD21^+^CD24^+^ | -23* (-48 / 38) | -29* (-48 / 38) | -22* (-47 / 27) | -11 (-47 / 10) | -21 (-42 / 6) | *0.005* |
| CD20^+^CD21^+^CD24^+^CD27^+^ | -24* (-47 / 36) | -26 (-44 / 36) | -20* (-47 / 27) | -13 (-47 / 11) | -24 (-41 / 7) | *0.02* |
| CD20^+^CD21^+^CD24^+^CD27^-^ | -25* (-45 / 40) | -30* (-45 / 28) | -24* (-45 / 40) | -10 (-45 / 7) | -15 (-37 / 26) | *0.03* |
| CD20^++^CD21^-^ | -25* (-60 / 24) | -27* (-51 / 11) | -23* (-60 / 24) | -12 (-51 / 6) | -24 (-45 / 3) | *0.006* |
| CD20^++^CD21^-^CD24^+^ | -16* (-45 / 12) | -27* (-45 / 12) | -15* (-45 / 12) | -18* (-45 / 3) | -29 (-45 / 3) | *0.002* |
| CD20^++^CD21^-^CD24^+^CD27^+^ | -13* (-45 / 21) | -20* (-45 / 21) | -12* (-45 / 14) | -11 (-45 / 4) | -32* (-45 / 3) | *0.006* |
| CD20^++^CD21^-^CD24^+^CD27^-^ | -45* (-45 / 30) | -45* (-45 / 4) | -45* (-45 / 30) | -1 (-45 / 5) | -45 (-45 / 15) | *0.001* |
| CD20^++^CD21^-^CD24^-^ | -18* (-46 / 64) | -25* (-45 / 29) | -7 (-46 / 64) | 2 (-45 / 39) | -13 (-45 / 8) | *0.09* |
| CD20^++^CD21^-^CD24^-^CD27^+^ | 0 (-45 / 143) | 0 (-45 / 38) | 0 (-45 / 143) | 6 (-45 / 34) | 0 (-45 / 18) | *0.44* |
| CD20^++^CD21^-^CD24^-^CD27^-^ | -45* (-45 / 34) | -45* (-45 / 27) | -6 (-45 / 34) | 1 (-45 / 46) | -45* (-45 / 11) | *0.04* |
| **IgG3^+^** | -32* (-65 / 62) | -39* (-58 / 62) | -26* (-65 / 62) | -1 (-64 / 21) | -16 (-45 / 25) | *0.005* |
| CD20^+^CD21^+^CD24^+^ | -31* (-57 / 70) | -36* (-48 / 70) | -29* (-57 / 53) | -2 (-56 / 22) | -17 (-42 / 12) | *0.01* |
| CD20^+^CD21^+^CD24^+^CD27^+^ | -25* (-54 / 95) | -28 (-46 / 95) | -20* (-54 / 46) | -3 (-50 / 33) | -14 (-41 / 19) | *0.14* |
| CD20^+^CD21^+^CD24^+^CD27^-^ | -30* (-50 / 49) | -30* (-46 / 48) | -31* (-50 / 49) | -12 (-56 / 16) | 1^#!^ (-37 / 24) | *<0.001* |
| CD20^++^CD21^-^ | -28* (-69 / 74) | -32* (-60 / 46) | -27* (-69 / 74) | 1 (-58 / 22) | -2 (-53 / 36) | *0.004* |
| CD20^++^CD21^-^CD24^+^ | -31* (-63 / 80) | -38* (-61 / 55) | -23* (-63 / 80) | -16 (-61 / 10) | -35* (-58 / 14) | *<0.001* |
| CD20^++^CD21^-^CD24^+^CD27^+^ | -26* (-46 / 99) | -31* (-46 / 99) | -13* (-46 / 92) | -7 (-45 / 16) | -38* (-46 / 33) | *0.004* |
| CD20^++^CD21^-^CD24^+^CD27^-^ | -45* (-46 / 61) | -37* (-45 / 18) | -45* (-46 / 61) | -14^!^ (-45 / 7) | -11 (-45 / 1) | *<0.001* |
| CD20^++^CD21^-^CD24^-^ | -19* (-64 / 38) | -10 (-64 / 35) | -20* (-64 / 38) | 4 (-64 / 34) | 4 (-65 / 59) | *0.08* |
| CD20^++^CD21^-^CD24^-^CD27^+^ | -9 (-45 / 107) | 0 (-45 / 93) | -10 (-45 / 107) | 2 (-45 / 69) | 6 (-45 / 268) | *0.60* |
| CD20^++^CD21^-^CD24^-^CD27^-^ | -17* (-45 / 38) | -12 (-45 / 31) | -17* (-45 / 38) | 4^#!^ (-45 / 25) | 5^#!^ (-45 / 29) | *0.05* |
| **IgG4^+^** | 0 (-46 / 56) | 1 (-46 / 56) | 0 (-46 / 24) | 2 (-45 / 12) | -25 (-45 / 15) | *0.74* |
| CD20^+^CD21^+^CD24^+^ | 0 (-45 / 56) | 1 (-44 / 56) | 0 (-45 / 25) | 2 (-45 / 12) | -27 (-45 / 16) | *0.72* |
| CD20^+^CD21^+^CD24^+^CD27^+^ | 1 (-46 / 52) | 2 (-40 / 52) | 1 (-46 / 30) | 4 (-45 / 15) | -24 (-45 / 20) | *0.57* |
| CD20^+^CD21^+^CD24^+^CD27^-^ | -23* (-45 / 36) | -20 (-45 / 36) | -23* (-45 / 29) | -37 (-45 / 21) | -17 (-45 / 3) | *0.05* |
| CD20^++^CD21^-^ | -45 (-45 / 43) | -45 (-45 / 42) | -45 (-45 / 43) | -24 (-45 / 16) | -45* (-45 / 11) | *0.13* |
| CD20^++^CD21^-^CD24^+^ | -45* (-45 / 48) | -45* (-45 / 48) | -45* (-45 / 39) | -45 (-45 / 13) | -45* (-45 / 5) | *0.02* |
| CD20^++^CD21^-^CD24^+^CD27^+^ | 0 (-45 / 81) | 0 (-45 / 81) | 0 (-45 / 37) | 0 (0 / 11) | 0 (0 / 6) | *0.66* |
| CD20^++^CD21^-^CD24^+^CD27^-^ | 0* (0 / 230) | 0* (0 / 0) | 0 (0 / 230) | 0 (0 / 29) | 0 (0 / 0) | *0.06* |
| CD20^++^CD21^-^CD24^-^ | 0 (0 / 152) | 0 (0 / 133) | 0 (0 / 152) | 0 (0 / 63) | 0 (0 / 98) | *0.86* |
| CD20^++^CD21^-^CD24^-^CD27^+^ | 0 (0 / 425) | 0 (0 / 248) | 0 (0 / 425) | 0 (0 / 66) | 0 (0 / 203) | *0.994* |
| CD20^++^CD21^-^CD24^-^CD27^-^ | 0* (0 / 0) | 0 (0 / 0) | 0* (0 / 0) | 0^#^ (0 / 51) | 0 (0 / 0) | *0.17* |
| **IgA1^+^** | -5 (-58 / 49) | -21 (-52 / 49) | 0 (-58 / 23) | -1 (-69 / 15) | -9 (-45 / 9) | *0.45* |
| CD20^+^CD21^+^CD24^+^ | -5* (-55 / 52) | -22 (-54 / 52) | -4 (-55 / 22) | -5 (-63 / 15) | -8 (-46 / 6) | *0.31* |
| CD20^+^CD21^+^CD24^+^CD27^+^ | -6 (-56 / 58) | -14 (-55 / 58) | -5 (-56 / 29) | 2 (-60 / 16) | -7 (-57 / 8) | *0.81* |
| CD20^+^CD21^+^CD24^+^CD27^-^ | -26* (-46 / 26) | -32 (-46 / 26) | -23* (-46 / 11) | -20 (-49 / 29) | -25 (-37 / 5) | *0.02* |
| CD20^++^CD21^-^ | 0 (-54 / 51) | -20 (-54 / 12) | 1 (-50 / 51) | 0 (-72 / 47) | -13 (-39 / 22) | *0.64* |
| CD20^++^CD21^-^CD24^+^ | 0 (-54 / 46) | -11 (-54 / 15) | 1 (-45 / 46) | -2 (-45 / 24) | 0 (-30 / 14) | *0.61* |
| CD20^++^CD21^-^CD24^+^CD27^+^ | 0 (-45 / 51) | -11 (-45 / 26) | 1 (-45 / 51) | 0 (-45 / 23) | 0 (-45 / 11) | *0.45* |
| CD20^++^CD21^-^CD24^+^CD27^-^ | -12* (-45 / 21) | -31 (-45 / 8) | -10 (-45 / 21) | -9 (-45 / 23) | -34 (-45 / 24) | *0.18* |
| CD20^++^CD21^-^CD24^-^ | -5 (-46 / 109) | -14 (-46 / 30) | -2 (-45 / 109) | -9 (-46 / 73) | -20 (-46 / 38) | *0.61* |
| CD20^++^CD21^-^CD24^-^CD27^+^ | 4 (-45 / 219) | 3 (-45 / 51) | 5 (-45 / 219) | 4 (-45 / 77) | -2 (-45 / 45) | *0.55* |
| CD20^++^CD21^-^CD24^-^CD27^-^ | -5 (-45 / 50) | -16 (-45 / 23) | 1 (-45 / 50) | -2 (-45 / 58) | -22 (-45 / 35) | *0.57* |
| **IgA2^+^** | -22* (-65 / 35) | -27 (-54 / 35) | -17* (-65 / 17) | -22 (-72 / 11) | -24 (-48 / 2) | *0.03* |
| CD20^+^CD21^+^CD24^+^ | -20* (-66 / 35) | -28 (-54 / 35) | -18* (-66 / 16) | -25 (-71 / 11) | -22 (-48 / 2) | *0.03* |
| CD20^+^CD21^+^CD24^+^CD27^+^ | -19* (-58 / 38) | -24 (-50 / 38) | -15* (-58 / 18) | -24 (-61 / 16) | -17 (-49 / 3) | *0.07* |
| CD20^+^CD21^+^CD24^+^CD27^-^ | -29* (-48 / 11) | -37* (-48 / 9) | -27* (-48 / 11) | -31* (-46 / 3) | -28 (-37 / 3) | *<0.001* |
| CD20^++^CD21^-^ | -11 (-45 / 69) | -13 (-45 / 69) | -11 (-45 / 56) | 3 (-45 / 24) | -16 (-45 / 23) | *0.78* |
| CD20^++^CD21^-^CD24^+^ | -16 (-45 / 39) | -10 (-45 / 22) | -21* (-45 / 39) | 0 (-45 / 37) | -33 (-45 / 30) | *0.30* |
| CD20^++^CD21^-^CD24^+^CD27^+^ | -17 (-45 / 37) | -2 (-45 / 21) | -45 (-45 / 37) | 2 (-45 / 24) | -45 (-45 / 17) | *0.66* |
| CD20^++^CD21^-^CD24^+^CD27^-^ | 0* (0 / 26) | 0* (0 / 0) | 0* (0 / 26) | 0 (0 / 51) | 0 (0 / 85) | *0.004* |
| CD20^++^CD21^-^CD24^-^ | 1 (-45 / 138) | -45 (-45 / 125) | 2 (-45 / 138) | -7 (-45 / 52) | -45 (-45 / 22) | *0.56* |
| CD20^++^CD21^-^CD24^-^CD27^+^ | 0 (-45 / 238) | 0 (-45 / 204) | 0 (-45 / 238) | 0^ (0 / 50) | -45*^#^"^!^ (-45 / 27) | *0.07* |
| CD20^++^CD21^-^CD24^-^CD27^-^ | 0 (-45 / 80) | 0* (-45 / 80) | 0 (-45 / 46) | 0 (-45 / 50) | -22* (-45 / 18) | *0.04* |
| **IgD^+^** | -3 (-45 / 665) | -10 (-45 / 665) | -1 (-45 / 125) | 0 (-45 / 10) | 3 (-45 / 35) | *0.75* |
| CD20^+^CD21^+^CD24^+^ | 0 (-45 / 729) | 0 (-45 / 729) | 0 (-45 / 140) | 1 (-45 / 11) | 4 (-45 / 36) | *0.81* |
| CD20^++^CD21^-^ | 0 (0 / 198) | 0 (0 / 198) | 0 (0 / 42) | 1 (0 / 15) | 5 (0 / 29) | *0.44* |
| CD20^++^CD21^-^CD24^+^ | 0* (0 / 91) | 0 (0 / 91) | 0* (0 / 60) | 0^#!^ (0 / 20) | 0 (0 / 16) | *0.10* |
| CD20^++^CD21^-^CD24^-^ | 0 (0 / 323) | 0 (0 / 323) | 0 (0 / 46) | 0 (0 / 22) | 0 (0 / 44) | *0.39* |
| **IgH^-^** | 1 (-58 / 82) | 1 (-55 / 51) | 0 (-58 / 82) | -1 (-47 / 13) | -6 (-51 / 27) | *0.86* |
| **PC** | -22* (-62 / 77) | -11 (-45 / 77) | -24* (-62 / 3) | -27* (-60 / 1) | -17 (-49 / 1) | *0.001* |
| Total PC CD20^+^ | -45* (-45 / 13) | -45* (-45 / 13) | -45* (-45 / 8) | -45* (-45 / 5) | -6 (-45 / 6) | *<0.001* |
| Total PC CD20^-^ | -35* (-58 / 81) | -25* (-49 / 81) | -36* (-58 / 3) | -38* (-58 / 1) | -19* (-52 / 1) | *<0.001* |
| Total PC CD138^+^ | -38* (-45 / 26) | -37* (-45 / 26) | -39* (-45 / 21) | -42* (-45 / 0) | -40* (-45 / 0) | *<0.001* |
| Total PC CD138^-^ | -32* (-59 / 112) | -30* (-52 / 112) | -36* (-59 / 4) | -40* (-59 / 1) | -19 (-51 / 2) | *<0.001* |
| **IgM^+^** | -22* (-45 / 66) | -14 (-45 / 66) | -27* (-45 / 13) | -28* (-45 / 2) | 1 (-45 / 21) | *0.001* |
| CD20^+^CD138^-^ | 0 (0 / 63) | 0 (0 / 47) | 0*" (0 / 63) | 0 (0 / 19) | 5 (0 / 95) | *0.04* |
| CD20^-^CD138^-^ | -45* (-45 / 85) | -37* (-45 / 85) | -45* (-45 / 10) | -45* (-45 / 2) | -22 (-45 / 21) | *<0.001* |
| CD20^-^CD138^+^ | 0* (-45 / 51) | -22* (-45 / 51) | 0* (-45 / 18) | 0 (-45 / 3) | -45 (-45 / 6) | *0.01* |
| **Total IgG^+^ PC** | -31* (-57 / 3) | -31* (-46 / 1) | -31* (-57 / 3) | -32* (-46 / 2) | -27* (-52 / 1) | *<0.001* |
| **IgG1^+^** | -45* (-45 / 3) | -45* (-45 / 1) | -36* (-45 / 3) | -45* (-45 / 2) | -38* (-45 / 1) | *<0.001* |
| CD20^+^CD138^-^ | 0* (-45 / 19) | 0* (0 / 10) | 0* (-45 / 19) | 0 (0 / 14) | 0* (0 / 0) | *0.002* |
| CD20^-^CD138^-^ | -45* (-45 / 5) | -45* (-45 / 1) | -45* (-45 / 5) | -45* (-45 / 3) | -39 (-45 / 2) | *<0.001* |
| CD20^-^CD138^+^ | 0* (-45 / 8) | 0 (-45 / 3) | 0* (-45 / 8) | 0 (-45 / 1) | 0 (-45 / 0) | *0.05* |
| **IgG2^+^** | -21* (-59 / 6) | -18 (-46 / 3) | -25* (-59 / 6) | -20* (-46 / 1) | -40* (-59 / 2) | *0.001* |
| CD20^+^CD138^-^ | 0* (0 / 77) | 0* (0 / 0) | 0* (0 / 77) | 0* (0 / 0) | 0 (0 / 0) | *<0.001* |
| CD20^-^CD138^-^ | -45* (-47 / 7) | -23* (-45 / 6) | -45* (-47 / 7) | -31* (-45 / 2) | -39* (-47 / 1) | *<0.001* |
| CD20^-^CD138^+^ | 0* (-45 / 31) | 0* (-45 / 11) | 0* (-45 / 31) | 0^ (-45 / 4) | -22 (-45 / 0) | *0.04* |
| **IgG3^+^** | 0* (0 / 62) | 0 (0 / 34) | 0* (0 / 62) | 0^#!^ (0 / 73) | 0 (0 / 0) | *0.01* |
| CD20^+^CD138^-^ | 0 (0 / 0) | 0 (0 / 0) | 0 (0 / 0) | 0 (0 / 0) | 0 (0 / 0) | *0.50* |
| CD20^-^CD138^-^ | 0* (0 / 78) | 0* (0 / 0) | 0* (0 / 78) | 0^#^ (0 / 87) | 0 (0 / 0) | *0.04* |
| CD20^-^CD138^+^ | 0* (0 / 0) | 0 (0 / 0) | 0 (0 / 0) | 0^#^ (0 / 93) | 0 (0 / 0) | *0.22* |
| **IgG4^+^** | 0 (0 / 17) | 0 (0 / 8) | 0 (0 / 17) | 0 (0 / 7) | 0 (0 / 3) | *0.44* |
| CD20^+^CD138^-^ | 0 (0 / 93) | 0 (0 / 0) | 0 (0 / 93) | 0 (0 / 0) | 0 (0 / 0) | *0.66* |
| CD20^-^CD138^-^ | 0 (0 / 393) | 0 (0 / 116) | 0 (0 / 393) | 0 (0 / 100) | 0 (0 / 0) | *0.69* |
| CD20^-^CD138^+^ | 0 (0 / 20) | 0 (0 / 20) | 0 (0 / 0) | 0 (0 / 15) | 0 (0 / 0) | *0.37* |
| **Total IgA^+^ PC** | -29* (-57 / 114) | -18* (-48 / 114) | -29* (-57 / 3) | -36* (-57 / 1) | -28* (-51 / 1) | *<0.001* |
| **IgA1^+^** | -26* (-55 / 145) | -18* (-45 / 145) | -26* (-55 / 6) | -33* (-55 / 1) | -17* (-52 / 0) | *0.001* |
| CD20^+^CD138^-^ | -45* (-45 / 28) | -45* (-45 / 12) | -45* (-45 / 28) | -45* (-45 / 4) | -45* (-45 / 1) | *<0.001* |
| CD20^-^CD138^-^ | -27* (-57 / 220) | -29* (-50 / 220) | -27* (-57 / 3) | -37* (-49 / 1) | -11 (-46 / 1) | *<0.001* |
| CD20^-^CD138^+^ | -45* (-45 / 55) | -35* (-45 / 55) | -45* (-45 / 39) | -45* (-45 / 2) | -31* (-45 / 0) | *<0.001* |
| **IgA2^+^** | -28* (-49 / 27) | -18* (-46 / 27) | -28* (-49 / 9) | -33* (-46 / 0) | -42* (-46 / 1) | *<0.001* |
| CD20^+^CD138^-^ | 0* (-45 / 11) | 0 (-45 / 7) | 0 (-45 / 11) | 0 (-45 / 12) | 0 (0 / 5) | *0.32* |
| CD20^-^CD138^-^ | -34* (-50 / 49) | -29* (-46 / 49) | -36* (-50 / 10) | -35* (-49 / 1) | -45* (-46 / 1) | *<0.001* |
| CD20^-^CD138^+^ | -45* (-45 / 114) | -45* (-45 / 2) | -45* (-45 / 114) | -45* (-45 / 4) | -45* (-45 / 1) | *<0.001* |
| **IgD^+^** | 0* (0 / 69) | 0 (0 / 69) | 0* (0 / 51) | 0 (0 / 3) | 0 (0 / 0) | *0.05* |
| CD20^+^CD138^-^ | 0 (0 / 0) | 0 (0 / 0) | 0 (0 / 0) | 0 (0 / 0) | 0 (0 / 0) | *0.28* |
| CD20^-^CD138^-^ | 0* (0 / 76) | 0 (0 / 76) | 0* (0 / 41) | 0 (0 / 3) | 0 (0 / 0) | *0.15* |
| CD20^-^CD138^+^ | 0 (0 / 81) | 0 (0 / 41) | 0 (0 / 81) | 0 (0 / 0) | 0 (0 / 0) | *0.35* |

Results expressed as median (range) normalized (vs HD) absolute cell count (cells/µl) percentile values, with median values and both the 5^th^ and 95^th^ percentiles of HD corresponding to 0, -45 and +45 values.

HD, healthy donor; Ig, immunoglobulin; MBC, memory B-cells; max, maximum; MGUS, monoclonal gammopathy of undetermined significance; min, minimum; PC, plasma cells; Pre-GC, per-germinal center B-cells; SMM, smoldering multiple myeloma; SWM, smoldering Waldenström’s macroglobulinemia. **p*<0.05 vs HD; ^#^*p*<0.05 vs MGUS; “*p*<0.05 vs IgM-MGUS; ^!^*p*<0.05 vs IgM-MGUS; ^*p*<0.05 vs SWM.

**Supplementary Table S3**. Distribution of the major populations of innate myeloid cells (A), TCD4+ cells (B), cytotoxic T/NK-cells (C) and both B-cells and plasma cells (D) altered in blood of monoclonal gammopathy of undetermined significance (MGUS) (n=55), smoldering multiple myeloma (SMM) (n=12) and smoldering Waldenström’s macroglobulinemia (SWM) (n=8) patients compared to aged-matched healthy adults (n=118).

| **A: Innate myeloid cells** | **MGUS** | **SMM** | **SWM** |
| --- | --- | --- | --- |
| **Eosinophils** | ↑ 6 *(0.01)* | = 1 *(0.31)* | ↑ 17 *(0.04)* |
| **Neutrophils** | = 6 *(0.67)* | ↓ -23 *(0.003)* | = 4 *(0.46)* |
| Mature neutrophils | = 6 *(0.67)* | ↓ -23 *(0.003)* | = 4 *(0.46)* |
| **Monocytes** | = -3 *(0.83)* | = -16 *(0.22)* | ↑ 39 *(<0.001)* |
| cMo | = 2 *(0.33)* | = -6 *(0.49)* | ↑ 47 *(<0.001)* |
| cMo CD62L^+^ | ↑ 7 *(0.01)* | = 5 *(0.41)* | ↑ 31 *(0.005)* |
| cMo CD62L^-^ | ↓ -23 *(0.04)* | ↓ -37 *(0.006)* | ↑ 25 *(<0.001)* |
| cMo FcεRI^-^ | = 7 *(0.14)* | = -5 *(0.98)* | ↑ 29 *(0.001)* |
| ncMo | ↓ -21 *(0.001)* | = -19 *(0.06)* | = 6 *(0.46)* |
| ncMo CD36^+^ | ↓ -20 *(<0.001)* | ↓ -24 *(0.02)* | = 7 *(0.64)* |
| ncMo CD36^-^ | ↓ -11 *(0.003)* | = -17 *(0.11)* | = 5 *(0.55)* |
| ncMo Slan^+^ | ↓ -18 *(<0.001)* | = -15 *(0.10)* | = -3 *(0.88)* |
| ncMo Slan^-^ | ↓ -17 *(0.004)* | ↓ -23 *(0.05)* | = 1 *(0.43)* |
| **Dendritic cells** | ↓ -20 *(0.03)* | = -23 *(0.45)* | = -12 *(0.36)* |
| mDC | ↓ -19 *(0.02)* | = -13 *(0.51)* | = -2 *(0.58)* |
| mDC CD1c^+^ | ↓ -18 *(0.03)* | = -13 *(0.55)* | = -3 *(0.64)* |
| mDC CD141^+^ | ↓ -16 *(<0.001)* | ↓ -27 *(0.04)* | = -13 *(0.26)* |
| pDC | = -14 *(0.11)* | = -20 *(0.11)* | ↓ -48 *(0.02)* |
| Axl^+^ DC | ↓ -16 *(0.02)* | ↓ -23 *(0.02)* | ↓ -28 *(0.03)* |
| **M-MDSC** | = 3 *(0.07)* | = -7 *(0.63)* | ↑ 12 *(0.02)* |
| **ILC** | ↓ -17 *(<0.001)* | = -9 *(0.24)* | = -9 *(0.57)* |
| ILC2 | ↓ -45 *(0.01)* | = -25 *(0.43)* | = -45 *(0.42)* |
| ILC3 | ↓ -15 *(<0.001)* | = -5 *(0.26)* | = -3 *(0.72)* |

Results expressed as median normalized (vs HD) absolute cell count (cells/µl) variation values, with median values of HD corresponding to 0.

cMo, classical monocytes; DC, dendritic cells; HD, healthy donor; mDC, myeloid dendritic cells; MGUS, monoclonal gammopathy of undetermined significance; M-MDSC, monocytic myeloid-derived suppressor cells; ncMo, non-classical monocytes; pDC, plasmacytoid dendritic cells; SMM, smoldering multiple myeloma; SWM, smoldering Waldenström’s macroglobulinemia;. ↓ significantly decreased vs HD. ↑ significantly increased vs HD. = not significantly altered vs HD.

**Supplementary Table S3** (continued).

| **B: TCD4^+^ cells** | **MGUS** | **SMM** | **SWM** |
| --- | --- | --- | --- |
| **T-cells** | ↓ -11 *(0.03)* | = 8 *(0.64)* | = 0 *(0.98)* |
| Tregs | ↓ -21 *(0.002)* | = -16 *(0.14)* | ↓ -34 *(0.03)* |
| TCD4^+^ Naive | ↓ -13 *(0.02)* | ↓ -22 *(0.04)* | = -24 *(0.14)* |
| Th2 | ↓ -23 *(0.008)* | = 1 *(0.83)* | = -25 *(0.07)* |
| Th22 | = 1 *(0.50)* | ↑ 16 *(0.004)* | = -17 *(0.20)* |
| Th1-17 | ↓ -18 *(0.001)* | = -9 *(0.44)* | = -10 *(0.28)* |
| TCD4^+^ CD183^+^CD194^+^CD196^+^CCR10^+^ | = 2 *(0.26)* | ↑ 12 *(0.03)* | = -11 *(0.47)* |
| TCD4^+^ CD183^+^CD194^+^CD196^-^CCR10^+^ | ↑ 7 *(0.01)* | ↑ 13 *(0.005)* | = 4 *(0.93)* |
| TCD4^+^ CD183^+^CD194^-^CD196^-^CCR10^+^ | = 1 *(0.31)* | ↑ 28 *(0.02)* | = 12 *(0.53)* |
| TCD4^+^ CD183^-^CD194^+^CD196^-^CCR10^+^ | ↑ 2 *(0.03)* | ↑ 7 *(0.03)* | = -18 *(0.13)* |

Results expressed as median normalized (vs HD) absolute cell count (cells/µl) variation values, with median values of HD corresponding to 0.

HD, healthy donor; ILC, innate lymphoid cells; MGUS, monoclonal gammopathy of undetermined significance; SMM, smoldering multiple myeloma; SWM, smoldering Waldenström’s macroglobulinemia; Th, helper T-cells; Treg, regulatory T-cells. ↓ significantly decreased vs HD. ↑ significantly increased vs HD. = not significantly altered vs HD.

**Supplementary Table S3** (continued).

| **C: Cytotoxic T/NK-cells** | **MGUS** | **SMM** | **SWM** |
| --- | --- | --- | --- |
| **Tγδ^+^ cells** | = -4 *(0.58)* | ↓ -12 *(0.05)* | ↓ -30 *(0.02)* |
| Total Tγδ^+^ cyGranzB^-^ | ↓ -21 *(0.01)* | = -24 *(0.15)* | ↓ -38 *(0.01)* |
| Tγδ^+^ TM | ↓ -21 *(0.04)* | = -27 *(0.27)* | ↓ -35 *(0.03)* |
| Total TCD8^+^ cyGranzB^+^ | ↑ 10 *(0.03)* | = 13 *(0.06)* | ↑ 33 *(0.008)* |
| Total TCD8^+^ cyGranzB^-^ | ↑ 10 *(<0.001)* | ↑ 10 *(0.03)* | ↑ 12 *(0.04)* |
| Total TCD8^+^ CD57^+^ | ↑ 9 *(0.008)* | = 15 *(0.06)* | ↑ 33 *(0.007)* |
| Total TCD8^+^ CD57^-^ | ↑ 11 *(<0.001)* | ↑ 12 *(0.02)* | ↑ 16 *(0.03)* |
| TCD8^+^ Naive | ↓ -12 *(0.007)* | ↓ -41 *(<0.001)* | = -28 *(0.09)* |
| TCD8^+^ TM | = -2 *(0.77)* | ↑ 19 *(0.05)* | = 6 *(0.32)* |
| TCD8^+^ EM | = 6 *(0.27)* | ↑ 13 *(0.04)* | ↑ 15 *(0.03)* |
| TCD8^+^ CD27^+^CD45RA^-^CD62L^+^cyGranzB^+^ | = 1 *(0.34)* | ↑ 7 *(0.02)* | = 1 *(0.36)* |
| TCD8^+^ CD27^+^CD45RA^-^CD62L^-^cyGranzB^+^ | = 0 *(0.34)* | ↑ 8 *(0.01)* | ↑ 11 *(0.007)* |
| **NK-cells** | ↓ -29 *(<0.001)* | ↓ -21 *(0.01)* | ↓ -25 *(0.03)* |
| Total NK cyGranzB^+^ | ↓ -26 *(<0.001)* | ↓ -18 *(0.03)* | ↓ -26 *(0.04)* |
| Total NK CD57^-^ | ↓ -35 *(<0.001)* | ↓ -29 *(0.002)* | ↓ -36 *(0.008)* |
| NK CD56^lo^ | ↓ -30 *(<0.001)* | ↓ -22 *(0.01)* | ↓ -26 *(0.03)* |

Results expressed as median normalized (vs HD) absolute cell count (cells/µl) variation values, with median values of HD corresponding to 0.

EM, effector memory; GranzB, Granzyme-B; HD, healthy donor; MGUS, monoclonal gammopathy of undetermined significance; SMM, smoldering multiple myeloma; SWM, smoldering Waldenström’s macroglobulinemia; TM, transitional memory. ↓ significantly decreased vs HD. ↑ significantly increased vs HD. = not significantly altered vs HD.

**Supplementary Table S3** (continued).

| **D: B-cells and plasma cells** | **MGUS** | **SMM** | **SWM** |  |
| --- | --- | --- | --- | --- |
| **B-cells** | ↓ -32 *(<0.001)* | ↓ -30 *(0.001)* | = -26 *(0.29)* |  |
| Pre-GC | ↓ -24 *(<0.001)* | ↓ -28 *(0.003)* | = -6 *(0.73)* |  |
| Immature | ↓ -18 *(0.009)* | = -17 *(0.15)* | = 1 *(0.72)* |  |
| Naive | ↓ -26 *(<0.001)* | ↓ -27 *(0.004)* | = -9 *(0.72)* |  |
| Naive CD5^+^ | ↓ -18 *(0.04)* | = 5 *(0.55)* | = 6 *(0.32)* |  |
| Naive CD5^-^ | ↓ -30 *(<0.001)* | ↓ -34 *(<0.001)* | = 17 *(0.33)* |  |
| MBC | ↓ -30 *(<0.001)* | ↓ -17 *(0.01)* | ↓ -34 *(0.01)* |  |
| Total MBC CD27^+^ | ↓ -22 *(0.004)* | = -15 *(0.17)* | = -26 *(0.14)* |  |
| Total MBC CD27^-^ | ↓ -33 *(<0.001)* | ↓ -26 *(0.04)* | = -23 *(0.06)* |  |
| Total MBC CD21^+^ | ↓ -29 *(<0.001)* | ↓ -18 *(0.01)* | ↓ -38 *(0.01)* |  |
| Total MBC CD21^-^ | ↓ -35 *(<0.001)* | ↓ -16 *(0.04)* | ↓ -30 *(0.04)* |  |
| IgMD^+^ | ↓ -38 *(<0.001)* | ↓ -35 *(<0.001)* | ↓ -41 *(0.004)* |  |
| IgG1^+^ | ↓ -13 *(0.02)* | = 4 *(0.40)* | = -4 *(0.41)* |  |
| IgG2^+^ | ↓ -22 *(<0.001)* | = -10 *(0.20)* | = -19 *(0.09)* |  |
| IgG3^+^ | ↓ -32 *(<0.001)* | = -1 *(0.52)* | = -16 *(0.27)* |  |
| IgA2^+^ | ↓ -22 *(0.005)* | = -22 *(0.10)* | = -24 *(0.05)* |  |
| **PC** | ↓ -22 *(0.001)* | ↓ -27 *(0.01)* | = -16 *(0.08)* |  |
| Total PC CD20^+^ | | ↓-45 *(<0.001)* | ↓ -45 *(0.004)* | = -6 *(0.37)* |
| Total PC CD20^-^ | | ↓ -35 *(<0.001)* | ↓ -38 *(<0.001)* | ↓ -19 *(0.04)* |
| Total PC CD138^+^ | | ↓ -38 *(<0.001)* | ↓ -42 *(0.002)* | ↓ -40 *(0.008)* |
| Total PC CD138^-^ | | ↓ -32 *(<0.001)* | ↓ -40 *(0.001)* | = -19 *(0.08)* |
| IgM^+^ | ↓ -22 *(<0.001)* | ↓ -28 *(0.02)* | = 1 *(0.69)* |  |
| Total PC IgG | ↓ -31 *(<0.001)* | ↓ -32 *(0.01)* | ↓ -27 *(0.03)* |  |
| IgG1^+^ | ↓ -45 *(<0.001)* | ↓ -45 *(0.001)* | ↓ -38 *(0.03)* |  |
| IgG2^+^ | ↓ -21 *(<0.001)* | ↓ -20 *(0.02)* | ↓ -40 *(0.01)* |  |
| IgG3^+^ | ↑ 0 *(0.001)* | = 0 *(0.87)* | = 0 *(0.11)* |  |
| Total PC IgA | ↓ -29 *(<0.001)* | ↓ -36 *(0.003)* | ↓ -28 *(0.01)* |  |
| IgA1^+^ | ↓ -26 *(<0.001)* | ↓ -33 *(0.004)* | ↓ -17 *(0.05)* |  |
| IgA2^+^ | ↓ -28 *(<0.001)* | ↓ -33 *(0.007)* | ↓ -42 *(0.003)* |  |
| IgD^+^ | ↑ 0 *(0.01)* | = 0 *(0.25)* | = 0 *(0.14)* |  |

Results expressed as median normalized (vs HD) absolute cell count (cells/µl) variation values, with median values of HD corresponding to 0.

HD, healthy donor; Ig, immunoglobulin; MBC, memory B-cells; MGUS, monoclonal gammopathy of undetermined significance; PC, plasma cells; Pre-GC, per-germinal center B-cells; SMM, smoldering multiple myeloma; SWM, smoldering Waldenström’s macroglobulinemia. ↓ significantly decreased vs HD. ↑ significantly increased vs HD. = not significantly altered vs HD.

**Supplementary Table S4.** Distribution of the major and minor populations of innate myeloid cells, TCD4^+^ cells, cytotoxic T/NK-cells and both B-cells and plasma cells in BM of monoclonal gammopathy of undetermined significance (MGUS) (n=36), smoldering multiple myeloma (SMM) (n=9) and smoldering Waldenström’s macroglobulinemia (SWM) (n=3) patients compared to aged-matched healthy adults (n=9).

|  | **MGUS** | **SMM** | **SWM** | ***p* value** | |
| --- | --- | --- | --- | --- | --- |
| **Eosinophils** | 8 (-57 / 412) | 17* (-8 / 66) | 5 (-13 / 63) | | *0.39* |
| **Neutrophils** | -175* (-514 / -2) | -196* (-267 / -104) | -154* (-717 / -68) | | *< 0.001* |
| **Monocytes** | 41* (-165 / 324) | 48* (7 / 96) | 23 (-16 / 65) | | *0.07* |
| **Other innate cells (ILC, DC and basophils)** | 34* (-24 / 128) | 18* (5 / 90) | 18 (-24 / 53) | | *0.12* |
| **Lymphocytes** | 51* (-31 / 208) | 80* (11 / 154) | 17 (4 / 28) | | *0.007* |
| **T-cells** | 30* (-61 / 125) | 51* (7 / 109) | 18 (-85 / 25) | | *0.02* |
| **CD4^+^CD8^-^** | 15 (-75 / 86) | 20^ (-38 / 53) | -53^#^ (-123 / 4) | | *0.04* |
| **CD8^+^CD4^-^** | 141* (-37 / 1119) | 443* (44 / 869) | 286 (-11 / 332) | | *0.001* |
| **CD4^-^CD8^-^** | 0 (-48 / 85) | -17 (-39 / 16) | -34 (-35 / -25) | | *0.22* |
| **Tγδ^+^ cells** | -2 (-55 / 84) | -26 (-43 / 12) | -41 (-45 / -32) | | *0.21* |
| CD3^hi^ | -28 (-53 / 40) | -38 (-48 / 1) | -43 (-53 / -42) | | *0.10* |
| CD5^+^ | -28 (-54 / 27) | -36 (-48 / 3) | -46* (-54 / -41) | | *0.05* |
| CD5^-^/^lo^ | 3 (0 / 172) | 0 (0 / 10) | 1 (0 / 6) | | *0.20* |
| CD3^+^ | 14 (-70 / 194) | 1 (-62 / 66) | 1 (-47 / 1) | | *0.83* |
| CD5^+^ | 18 (-98 / 328) | 5 (-83 / 75) | -46 (-49 / 4) | | *0.69* |
| CD5^-^/^lo^ | 3 (-53 / 57) | -16 (-48 / 30) | 3 (-30 / 10) | | *0.85* |
| **Tγδ^-^** | -1 (-52 / 486) | 99* (-18 / 395) | -4 (-45 / 205) | | *0.20* |
| **B-cells** | 24 (-72 / 238) | 33 (-69 / 124) | 30 (-84 / 118) | | *0.51* |
| **Precursors** | 44* (-54 / 467) | 27 (-46 / 232) | 36 (-55 / 338) | | *0.17* |
| **Mature** | 9 (-60 / 209) | 38 (-53 / 84) | 9 (-63 / 24) | | *0.77* |
| smIg Kappa | 12 (-67 / 259) | 19 (-51 / 115) | -5 (-67 / 24) | | *0.75* |
| smIg Lambda | 6 (-53 / 160) | 41 (-56 / 79) | 24 (-58 / 24) | | *0.80* |
| **NK-cells** | 18* (-63 / 266) | 37* (-2 / 92) | 12 (-29 / 28) | | *0.07* |
| CD56^hi^ | 52* (-52 / 251) | 181*^#^ (-13 / 794) | 91 (-9 / 413) | | *0.006* |
| **PC** | 44* (-37 / 319) | 9 (-47 / 175) | 2 (-23 / 74) | | *0.05* |

Results expressed as median (range) normalized (vs normal BM) percentage percentile values, with median values and both the 5^th^ and 95^th^ percentiles of HD corresponding to 0, -45 and +45 values.

DC, dendritic cells; HD, healthy donor; ILC, innate lymphoid cells; max, maximum; MGUS, monoclonal gammopathy of undetermined significance; min, minimum; PC, plasma cells; sm, surface membrane; SMM, smoldering multiple myeloma; SWM, smoldering Waldenström’s macroglobulinemia. **p*<0.05 vs HD; ^#^*p*<0.05 vs MGUS; ^*p*<0.05 vs SWM.

**Supplementary Table S5.** Distribution of the major populations of innate myeloid cells, cytotoxic T/NK-cells and both B-cells and plasma cells altered in BM of monoclonal gammopathy of undetermined significance (MGUS) (n=36), smoldering multiple myeloma (SMM) (n=9) and smoldering Waldenström’s macroglobulinemia (SWM) (n=3) patients compared to aged-matched healthy adults (n=9).

|  | **MGUS** | **SMM** | **SWM** |  |
| --- | --- | --- | --- | --- |
| **Neutrophils** | ↓ -175 *(0.001)* | ↓ -196 *(0.001)* | ↓ -154 *(0.009)* | |
| **Monocytes** | ↑ 41 *(0.02)* | ↑ 48 *(0.006)* | = 23 *(0.86)* | |
| **Other leucocytes (ILC, DC, basophils)** | ↑ 34 *(0.03)* | ↑ 18 *(0.02)* | = 18 *(0.60)* | |
| **Lymphocytes** | ↑ 51 *(0.006)* | ↑ 80 *(0.001)* | = 17 *(0.28)* | |
| **T-cells** | ↑ 30 *(0.02)* | ↑ 51 *(0.003)* | = 18 *(1.00)* | |
| CD8^+^CD4^-^ | ↑ 141 *(0.001)* | ↑ 443 *(0.001)* | = 286 *(0.21)* | |
| Tγδ^-^ | = -1 *(0.32)* | ↑ 99 *(0.01)* | = -4 *(1.00)* | |
| Precursor B-cells | ↑ 44 *(0.03)* | = 27 *(0.11)* | = 36 *(0.73)* | |
| **NK-cells** | ↑ 18 *(0.04)* | ↑ 37 *(0.02)* | = 12 *(0.60)* | |
| CD56^hi^ | ↑ 52 *(0.01)* | ↑ 181 *(0.001)* | = 91 *(0.15)* | |
| **PC** | ↑ 44 *(0.006)* | = 9 *(0.30)* | = 2 *(0.48)* | |

Results expressed as median normalized (vs normal BM) percentage variation values, with median values of HD corresponding to 0.

DC, dendritic cells; ILC, innate lymphoid cells; MGUS, monoclonal gammopathy of undetermined significance; PC, plasma cells; SMM, smoldering multiple myeloma; SWM, smoldering Waldenström’s macroglobulinemia. ↓ significantly decreased vs normal BM. ↑ significantly increased vs normal BM. = not significantly altered vs normal BM.

**Supplementary Table S6**. Distribution of the major and minor populations of innate myeloid cells (A), TCD4^+^ cells (B), cytotoxic T/NK-cells (C) and both B-cells and plasma cells (D) with statistical differences in blood between IgM-monoclonal gammopathy of undetermined significance (MGUS) (n=18) and/or non-IgM MGUS (n=37) vs smoldering multiple myeloma (SMM) (n=12) and smoldering Waldenström’s macroglobulinemia (SWM) (n=8).

| **A: Innate myeloid cells** | **IgM-MGUS** | **non-IgM-MGUS** | **SMM** | **SWM** | ***p* value** |
| --- | --- | --- | --- | --- | --- |
| **Leucocytes** | 1 (-42 / 43) | 6 (-51 / 38) | -14^!^^ (-19 / 22) | 10" (-22 / 23) | *0.18* |
| **Neutrophils** | -4 (-82 / 34) | 10 (-88 / 48) | -23*^#!^ (-27 / 6) | 4 (-66 / 21) | *0.02* |
| Mature neutrophils | -4 (-82 / 34) | 10 (-88 / 48) | -23*^#!^ (-28 / 6) | 4 (-66 / 21) | *0.02* |
| **Monocytes** | -10 (-44 / 74) | 4 (-55 / 60) | -16^ (5 / 41) | 39*^#^"^!^ (3 / 101) | *0.003* |
| **cMo** | -7 (-35 / 113) | 4 (-45 / 61) | -6^ (3 / 40) | 47*^#^"^!^ (10 / 96) | *0.004* |
| cMo CD62L^-^FcεRI^+^ | -20 (-43 / 66) | -29* (-44 / 113) | -15^ (7 / 10) | 11*^#^"^!^ (1 / 35) | *0.004* |
| cMo CD62L^-^FcεRI^-^ | -22 (-43 / 76) | -19 (-49 / 49) | -26^ (3 / 25) | 21*^#^"^!^ (9 / 67) | *0.003* |
| cMo CD62L^-^ | -26 (-50 / 110) | -23 (-54 / 49) | -37*^ (2 / 25) | 25*^#^"^!^ (12 / 64) | *<0.001* |
| cMo FcεRI^+^ | -8 (-43 / 42) | -20 (-44 / 35) | -16^ (8 / 16) | 5^!^ (-18 / 34) | *0.24* |
| cMo FcεRI^-^ | 1 (-68 / 65) | 8 (-53 / 99) | -5^ (24 / 24) | 29*^#^"^!^ (5 / 85) | *0.009* |
| **ncMo** | -23* (-58 / 34) | -14* (-49 / 353) | -19 (12 / 27) | 6^#^" (-32 / 136) | *0.004* |
| ncMo CD36^+^Slan^-^ | -30* (-50 / 9) | -16* (-47 / 37) | -22* (20 / 19) | 9^#^"^!^ (-34 / 44) | *0.002* |
| ncMo CD36^+^ | -32* (-53 / 8) | -18* (-49 / 36) | -24* (17 / 18) | 7^#^"^!^ (-39 / 43) | *<0.001* |
| ncMo Slan^-^ | -20* (-48 / 31) | -17* (-42 / 429) | -23*^ (15 / 30) | 1^#^"^!^ (-24 / 44) | *0.006* |
| **M-MDSC** | 5 (-11 / 42) | 3 (-35 / 58) | -7"^ (16 / 14) | 12*^#!^ (4 / 26) | *0.02* |

Results expressed as median (range) normalized (vs HD) absolute cell count (cells/µl) percentile values, with median values and both the 5^th^ and 95^th^ percentiles of HD corresponding to 0, -45 and +45 values.

cMo, classical monocytes; HD, healthy donor; max, maximum; MGUS, monoclonal gammopathy of undetermined significance; min, minimum; M-MDSC, monocytic myeloid-derived suppressor dells; ncMo, non-classical monocytes; SMM, smoldering multiple myeloma; SWM, smoldering Waldenström’s macroglobulinemia. **p*<0.05 vs HD; ^#^*p*<0.05 vs IgM-MGUS; ^!^*p*<0.05 vs non-IgM-MGUS; ^*p*<0.05 vs SWM.

**Supplementary Table S6** (continued).

| **B: TCD4^+^ cells** | **IgM-MGUS** | **Non-IgM-MGUS** | **SMM** | **SWM** | ***p value*** | |
| --- | --- | --- | --- | --- | --- | --- |
| TFH CD183^+^CD194^+^CD196^-^CCR10^-^ | -6 (-48 / 99) | 4 (-50 / 47) | 4 (-31 / 28) | -21^#!^ (-59 / 12) | | *0.30* |
| TFH CD183^-^CD194^-^CD196^+^CCR10^-^ | -9 (-52 / 29) | -18* (-54 / 30) | 0^#!^ (-30 / 40) | -22 (-40 / 55) | | *0.04* |
| Treg Th22-like | 1 (-33 / 95) | -1 (-58 / 87) | 3^ (-27 / 87) | -25*^#^" (-53 / 2) | | *0.13* |
| Treg CD183^+^CD194^+^CD196^-^CCR10^+^ | 3 (-45 / 27) | 4 (-45 / 54) | 6^ (-16 / 75) | -13^#^" (-45 / 9) | | *0.14* |
| Treg CD183^+^CD194^+^CD196^+^CCR10^-^ | -11 (-59 / 95) | -21 (-55 / 75) | -15 (-37 / 11) | -39*" (-53 / 38) | | *0.07* |
| **Th22** | 2 (-53 / 67) | 0 (-52 / 64) | 16*^#!^^ (-12 / 85) | -17 (-53 / 34) | | *0.03* |
| Th22 EM | 7 (-39 / 114) | 1 (-47 / 40) | 25*^#!^^ (-17 / 93) | -8 (-53 / 69) | | *0.003* |
| CD183^+^CD194^-^CD196^-^CCR10^+^ CM | 0 (0 / 48) | 0 (0 / 155) | 22*^#!^ (0 / 67) | 6 (0 / 18) | | *0.11* |
| CD183^+^CD194^-^CD196^-^CCR10^+^ TM | 0* (0 / 0) | 0 (0 / 178) | 8*^#^"^!^ (0 / 176) | 0 (0 / 52) | | *0.01* |
| CD183^-^CD194^-^CD196^+^CCR10^-^ EM | 0 (-45 / 47) | 0 (-45 / 37) | 0^!^ (0 / 113) | 7^#^ (0 / 36) | | *0.15* |
| **CD183^-^CD194^+^CD196^-^CCR10^+^** | 1 (-52 / 57) | 3* (-64 / 122) | 7*^ (-21 / 216) | -18^#!^ (-43 / 17) | | *0.02* |
| CD183^-^CD194^+^CD196^-^CCR10^+^ CM | 0 (-40 / 92) | 3 (-51 / 152) | 8*^ (-24 / 209) | -21^#!^ (-49 / 26) | | *0.01* |
| CD183^-^CD194^+^CD196^-^CCR10^+^ TM | 0 (0 / 32) | 10* (0 / 109) | 3^ (-45 / 306) | 0^#^"^!^ (-45 / 17) | | *<0.001* |

Results expressed as median (range) normalized (vs HD) absolute cell count (cells/µl) percentile values, with median values and both the 5^th^ and 95^th^ percentiles of HD corresponding to 0, -45 and +45 values.

CM, central memory; EM, effector memory; HD, healthy donor; HPC, hematopoietic progenitor cells; Ig, immunoglobulin; iMo, intermediate monocytes; MBC, memory B-cells; mDC, myeloid dendritic cells; max, maximum; MGUS, monoclonal gammopathy of undetermined significance; min, minimum; SMM, smoldering multiple myeloma; SWM, smoldering Waldenström’s macroglobulinemia; TFH, follicular helper T-cells; Th, helper T-cells; TM, transitional memory; Treg, regulatory T-cells. **p*<0.05 vs HD; ^#^*p*<0.05 vs IgM-MGUS; ^!^*p*<0.05 vs non-IgM-MGUS; ^*p*<0.05 vs SWM.

**Supplementary Table S6** (continued).

| **C: Cytotoxic T/NK-cells** | | **IgM-MGUS** | **Non-IgM-MGUS** | **SMM** | **SWM** | ***p* value** |
| --- | --- | --- | --- | --- | --- | --- |
| **Tγδ^+^ cells** | | -5 (-51 / 16) | -4 (-54 / 19) | -12* (-50 / 1) | -30*^#!^ (-51 / -1) | *0.08* |
| Total TCD8^+^ CD57^+^ | | 6 (-39 / 53) | 10* (-46 / 82) | 15 (-38 / 110) | 33*^#^" (-35 / 99) | *0.005* |
| **TCD8^+^ Naive** | | -9 (-63 / 60) | -16* (-62 / 45) | -41*^#!^ (-59 / 35) | -28 (-50 / 30) | *0.001* |
| TCD8^+^ Naive cyGranzB^-^CD57^-^ | | -10 (-63 / 60) | -15* (-62 / 43) | -42*^#!^ (-59 / 35) | -28 (-51 / 30) | *0.001* |
| TCD8^+^ EM cyGranzB^-^CD57^-^ | | -15 (-54 / 56) | -4 (-62 / 86) | 10 (-48 / 422) | 12*^#^"^!^ (-18 / 238) | *0.07* |
| TCD8+ EM cyGranzB+CD57- | 1 (-45 / 83) | 2 (-45 / 129) | 22*"^#^ (-38 / 1609) | 6 (-24 / 325) | *0.03* |  |
| CD27^+^CD45RA^-^CD62L^+^cyGranzB^+^CD57^-^ | | 1 (-44 / 65) | -2 (-53 / 91) | 7*^#!^ (-31 / 480) | 0 (-36 / 124) | *0.21* |
| **CD27^+^CD45RA^-^CD62L^-^cyGranzB^+^** | | -2 (-45 / 97) | 1 (-43 / 144) | 8* (-33 / 71) | 11*^#^"^!^ (-1 / 99) | *0.02* |
| CD27^+^CD45RA^-^CD62L^-^cyGranzB^+^CD57^-^ | | -1 (-49 / 62) | 1 (-44 / 240) | 10*"^#^ (-27 / 98) | 8 (-22 / 112) | *0.04* |
| CD27^+^CD45RA^-^CD62L^-^cyGranzB^+^CD57^+^ | | 1 (-42 / 80) | 1 (-34 / 36) | 5*^#!^ (-24 / 46) | 7* (-10 / 49) | *0.003* |
| NK CD56^hi^ cyGranzB^-^CD57^-^ | | 4 (-51 / 32) | -12 (-76 / 60) | 9^!^ (-21 / 42) | 4 (-62 / 25) | *0.23* |

Results expressed as median (range) normalized (vs HD) absolute cell count (cells/µl) percentile values, with median values and both the 5^th^ and 95^th^ percentiles of HD corresponding to 0, -45 and +45 values.

cy, cytoplasmic; EM, effector memory; GranzB, Granzyme-B; HD, healthy donor; max, maximum; MGUS, monoclonal gammopathy of undetermined significance; min, minimum; SMM, smoldering multiple myeloma; SWM, smoldering Waldenström’s macroglobulinemia. **p*<0.05 vs HD; ^#^*p*<0.05 vs IgM-MGUS; ^!^*p*<0.05 vs non-IgM-MGUS; ^*p*<0.05 vs SWM.

**Supplementary Table S6** (continued).

| **D: B-cells and plasma cells** | **IgM-MGUS** | **Non-IgM-MGUS** | **SMM** | **SWM** | ***p value*** |
| --- | --- | --- | --- | --- | --- |
| **MBC IgG1^+^** | -26 (-57 / 61) | -12* (-66 / 37) | 4^!^ (-65 / 27) | -4 (-57 / 19) | *0.10* |
| MBC IgG1^+^CD20^++^CD21^-^ | -15* (-63 / 17) | -11* (-63 / 34) | 8^#^"^!^ (-60 / 34) | -12 (-51 / 57) | *0.02* |
| MBC IgG1^+^CD20^++^CD21^-^CD24^+^ | -28* (-59 / 11) | -21* (-51 / 19) | 0^#^"^!^ (-55 / 22) | -25 (-46 / 11) | *0.003* |
| MBC IgG1^+^CD20^++^CD21^-^CD24^+^CD27^+^ | -22* (-50 / 16) | -14 (-43 / 32) | -4" (-46 / 30) | -28 (-41 / 14) | *0.05* |
| MBC IgG1^+^CD20^++^CD21^-^CD24^+^CD27^-^ | -28* (-45 / 8) | -23* (-45 / 25) | 0^#^" (-45 / 57) | -20 (-25 / 2) | *0.003* |
| MBC IgG1^+^CD20^++^CD21^-^CD24^-^ | -24 (-63 / 89) | -15 (-60 / 83) | 13^#^"^!^ (-59 / 58) | -5 (-57 / 321) | *0.06* |
| MBC IgG1^+^CD20^++^CD21^-^CD24^-^CD27^+^ | -17 (-46 / 65) | 0 (-46 / 273) | 16*^#^"^!^ (-33 / 98) | -8 (-46 / 236) | *0.18* |
| MBC IgG1^+^CD20^++^CD21^-^CD24^-^CD27^-^ | -13 (-57 / 56) | -12 (-69 / 29) | 5^#^"^!^ (-40 / 41) | 3 (-58 / 173) | *0.04* |
| MBC IgG3^+^CD20^+^CD21^+^CD24^+^CD27^-^ | -30* (-46 / 48) | -31* (-50 / 49) | -12 (-56 / 16) | 1^#!^ (-37 / 24) | *<0.001* |
| MBC IgG3^+^CD20^++^CD21^-^CD24^+^CD27^-^ | -37* (-45 / 18) | -45* (-46 / 61) | -14^!^ (-45 / 7) | -11 (-45 / 1) | *<0.001* |
| MBC IgG3^+^CD20^++^CD21^-^CD24^-^CD27^-^ | -12 (-45 / 31) | -17* (-45 / 38) | 4^#!^ (-45 / 25) | 5^#!^ (-45 / 29) | *0.05* |
| MBC IgA2^+^ CD20^++^CD21^-^CD24^-^CD27^+^ | 0 (-45 / 204) | 0 (-45 / 238) | 0^ (0 / 50) | -45*^#^"^!^ (-45 / 27) | *0.07* |
| MBC IgD^+^ CD20^++^CD21^-^CD24^+^ | 0 (0 / 91) | 0* (0 / 60) | 0^#!^ (0 / 20) | 0 (0 / 16) | *0.10* |
| PC IgM^+^ CD20^+^CD138^-^ | 0 (0 / 47) | 0*" (0 / 63) | 0 (0 / 19) | 5 (0 / 95) | *0.04* |
| **PC IgG3^+^** | 0 (0 / 34) | 0* (0 / 62) | 0^#!^ (0 / 73) | 0 (0 / 0) | *0.01* |

Results expressed as median (range) normalized (vs HD) absolute cell count (cells/µl) percentile values, with median values and both the 5^th^ and 95^th^ percentiles of HD corresponding to 0, -45 and +45 values.

HD, healthy donor; Ig, immunoglobulin; MBC, memory B-cells; max, maximum; MGUS, monoclonal gammopathy of undetermined significance; min, minimum; PC, plasma cells; SMM, smoldering multiple myeloma; SWM, smoldering Waldenström’s macroglobulinemia. **p*<0.05 vs HD; ^#^*p*<0.05 vs IgM-MGUS; ^!^*p*<0.05 vs non-IgM-MGUS; ^*p*<0.05 vs SWM.
